# RNAseq Analyses Identify Tumor Necrosis Factor-Mediated Inflammation as a Major Abnormality in ALS Spinal Cord

**DOI:** 10.1371/journal.pone.0160520

**Published:** 2016-08-03

**Authors:** David G. Brohawn, Laura C. O’Brien, James P. Bennett

**Affiliations:** 1 Parkinson’s Disease Research Center, Virginia Commonwealth University, Richmond, Virginia, United States of America; 2 Department of Human Genetics, Virginia Commonwealth University, Richmond, Virginia, United States of America; 3 Department of Physiology and Biophysics, Virginia Commonwealth University, Richmond, Virginia, United States of America; 4 Department of Neurology; Virginia Commonwealth University, Richmond, Virginia, United States of America; 5 Neurodegeneration Therapeutics, Inc., Charlottesville, Virginia, United States of America; Inserm, FRANCE

## Abstract

ALS is a rapidly progressive, devastating neurodegenerative illness of adults that produces disabling weakness and spasticity arising from death of lower and upper motor neurons. No meaningful therapies exist to slow ALS progression, and molecular insights into pathogenesis and progression are sorely needed. In that context, we used high-depth, next generation RNA sequencing (RNAseq, Illumina) to define gene network abnormalities in RNA samples depleted of rRNA and isolated from cervical spinal cord sections of 7 ALS and 8 CTL samples. We aligned >50 million 2X150 bp paired-end sequences/sample to the hg19 human genome and applied three different algorithms (*Cuffdiff2*, *DEseq2*, *EdgeR)* for identification of differentially expressed genes (DEG’s). Ingenuity Pathways Analysis (IPA) and Weighted Gene Co-expression Network Analysis (WGCNA) identified inflammatory processes as significantly elevated in our ALS samples, with tumor necrosis factor (TNF) found to be a major pathway regulator (IPA) and TNFα-induced protein 2 (TNFAIP2) as a major network “hub” gene (WGCNA). Using the oPOSSUM algorithm, we analyzed transcription factors (TF) controlling expression of the nine DEG/hub genes in the ALS samples and identified TF’s involved in inflammation (NFkB, REL, NFkB1) and macrophage function (NR1H2::RXRA heterodimer). Transient expression in human iPSC-derived motor neurons of TNFAIP2 (also a DEG identified by all three algorithms) reduced cell viability and induced caspase 3/7 activation. Using high-density RNAseq, multiple algorithms for DEG identification, and an unsupervised gene co-expression network approach, we identified significant elevation of inflammatory processes in ALS spinal cord with TNF as a major regulatory molecule. Overexpression of the DEG TNFAIP2 in human motor neurons, the population most vulnerable to die in ALS, increased cell death and caspase 3/7 activation. We propose that therapies targeted to reduce inflammatory TNFα signaling may be helpful in ALS patients.

## Introduction

Amyotrophic lateral sclerosis (ALS) is a disease characterized by degeneration of upper and lower motor neurons (MNs) in the brainstem and spinal cord. Average life expectancy after diagnosis is between 2–5 years, and current treatment (riluzole) extends life by only a few months.

~5% of ALS occurs in families (fALS), with 68% of European fALS cases explained by mutations in 9 loci (as of 2014). However, only 11% of European sporadic ALS (sALS) patients are accounted for by mutations in these loci [1]. Sporadically occurring ALS (sALS) represents ~95% of all ALS cases [2] and is widely thought to have both polygenic and environmental contributions [3, 4]. “Agnostic” exploratory association studies have been adopted to elucidate the genetic contributions to sALS [1, 5, 6].

Gene expression studies identify differentially expressed genes (DEG’s), which are genes with statistically significantly changed expression values between groups. Over-representation analyses can be used to infer their likely biological relevance. These analyses detect statistically significant associations between an input list of genes compared to predefined groups of genes known to influence various cellular processes

Heath et al [6] published a comprehensive review of ALS gene expression studies dating back to 2001. Tissue types compared between ALS and control samples included: human bicep, human lymphocytes, rodent gastrocnemius, human or rodent spinal tissue containing disease-vulnerable MNs, and isolated spinal MNs. There was a broad range in the number (14 to 1,182) and identity of ALS group-specific DEG’s discovered in each study.

Interestingly, a recurrent set of associated cellular processes emerged across over-representation analyses using these sets of DEGs. These included oxidative stress, mitochondrial dysfunction, apoptosis, cytoskeletal architecture, inflammation, RNA processing, and protein aggregation. Separate molecular biology assays revealed increased oxidative damage [7–11], abnormal mitochondrial morphology [12–18], and elevated inflammation [19–33] in various ALS tissues from human patients and fALS rodents. Increased inflammatory tumor necrosis factor (TNF) signaling [28–33] in ALS tissues may have therapeutic relevance, as it known to carry out cell fate decisions that may contribute to MN death [34]. Taken together, it is likely aberrations in these processes contribute to ALS onset, progression, and symptoms.

Exploratory gene expression studies comparing disease and control groups allow us to form novel hypotheses about what genes may functionally impact disease pathology. Candidate gene selection for downstream molecular biology experiments is rarely trivial, as these high throughput experiments often yield considerable options. Commonly, researchers select a candidate gene that 1) was identified as a DEG, 2) was in a group of DEG’s associated with cellular processes relevant to disease pathology, and 3) has known structural or functional properties plausibly connecting it to disease features.

Systems-level gene co-expression network analyses provide separate criteria for candidate gene selection that can be used in conjunction with DEG analysis results. These analyses typically follow a step-wise process that involves 1) prediction of interconnected gene networks using all samples’ gene expression measurements, 2) identification of networks statistically associated with phenotypic traits (i.e. disease status), 3) over-representation analyses to identify cellular processes associated with these networks, and 4) identification of highly connected “hub” genes predicted to organize each prioritized network’s activity.

This approach has identified networks associated with a polygenic trait and plausibly related cellular processes, and several of these networks’ hub genes were already associated with the trait studied using separate molecular biology techniques [35, 36]. Arguably more compelling, Horvath et al showed reducing expression of ASPM (a hub gene identified in a network associated with glioblastoma and mitosis) via siRNA significantly reduced proliferation rates in glioblastoma tumor cells *in vitro* [37].

In this study, we gathered >50 million 2X150 RNA-Sequencing reads in 15 postmortem spinal section tissues (7 ALS patients and 8 neurologically healthy controls) to characterize transcriptome-wide gene expression differences specific to our ALS group. We aligned reads to the hg19 reference genome using Bowtie2/Tophat2 [38]. We next performed three DEG analyses, a weighted gene co-expression network analysis, over-representation analyses of prioritized gene sets, and molecular assays testing a candidate hub gene’s potential to increase motor neuron death.

74 DEGs (56 up-regulated and 18 down-regulated) were identified in common across three analyses (Cuffdiff2, EdgeR, and DeSeq2, [39–41]) at an FDR of 0.10. Our gene network analysis (WGCNA, [42]) identified 37 different gene networks, and 2 were positively correlated with disease status at a p-value <0.01. QIAGEN’s Ingenuity Pathway Analysis (IPA®, QIAGEN Redwood City, www.qiagen.com/ingenuity), an over-representation analysis tool, revealed the 56 DEG’s up-regulated in the ALS group and genes comprising the gene network most strongly correlated to disease status were both associated with numerous inflammatory processes. Additionally, tumor necrosis factor (TNF) was identified as an "activated" upstream regulator in both gene groups. We prioritized this gene network for candidate gene selection, as 1) it was associated with disease status and 2) inflammatory processes and TNF signaling were recurrently associated with ALS pathology in previous literature [19–33]

To identify “hub” genes, we used three WGCNA metrics calculated for every gene within this network. We identified 12 “hub” genes (out of 495 total network genes) with scores in the top quartile for all three metrics. 9 of these genes were separately identified as up-regulated DEG’s in the ALS group across the three DEG analyses. TNFAIP2, a gene that encodes an intracellular protein of the tumor necrosis factor family, was one of them.

Based on these findings and previous literature, we hypothesized TNFAIP2 functionally mediates motor neuron death via TNF signaling in motor neurons. To test this, we compared cell viability and activated caspase 3/7 levels in human iPSC-derived motor neurons that overexpressed TNFAIP2-GFP or GFP for 24 hours. We show motor neurons that overexpressed TNFAIP2-GFP had decreased cell viability and increased activated caspase 3/7 levels compared to those overexpressing GFP alone. Taken together, these results suggest TNF signaling may play an important role in ALS pathology and reducing its activity could be of therapeutic relevance.

## Results

### Sequencing Metrics

We collected >50 million 2X150 RNA-Sequencing reads per sample in 15 postmortem spinal cord samples (7 ALS and 8 neurologically healthy controls). The following are averaged metrics compiled using Picard’s CollectRNASeqMetrics (http://picard.sourceforge.net): 68,613,940 2X150 raw sequencing read pairs, 20,584,182,060 total sequencing bases, 65.62% of bases that passed filter and aligned to the hg19 reference genome, 29.01% of aligned bases to rRNA/tRNA/mitochondrial RNA species, 33.23% of aligned bases to mRNA species, and 37.76% of aligned bases to intronic/intergenic species. Individual samples metrics can be found in S1 Fig.

### Presence of known ALS mutations and SOD1 transcription

As of 2014, 11% of sALS in Caucasians were explained by causal mutations in 9 different loci. Causal fALS mutations have been identified in at least 13 other genes, and may account for additional sALS cases [1, 43]. Genome Analysis Toolkit (GATK, [44]) provides a bioinformatics pipeline (https://www.broadinstitute.org/gatk/guide/article?id=3891) that identifies sequencing variants in the form of single nucleotide variants (SNVs), insertions, and deletions using input sequencing reads [44, 45].

**Fig 1:**
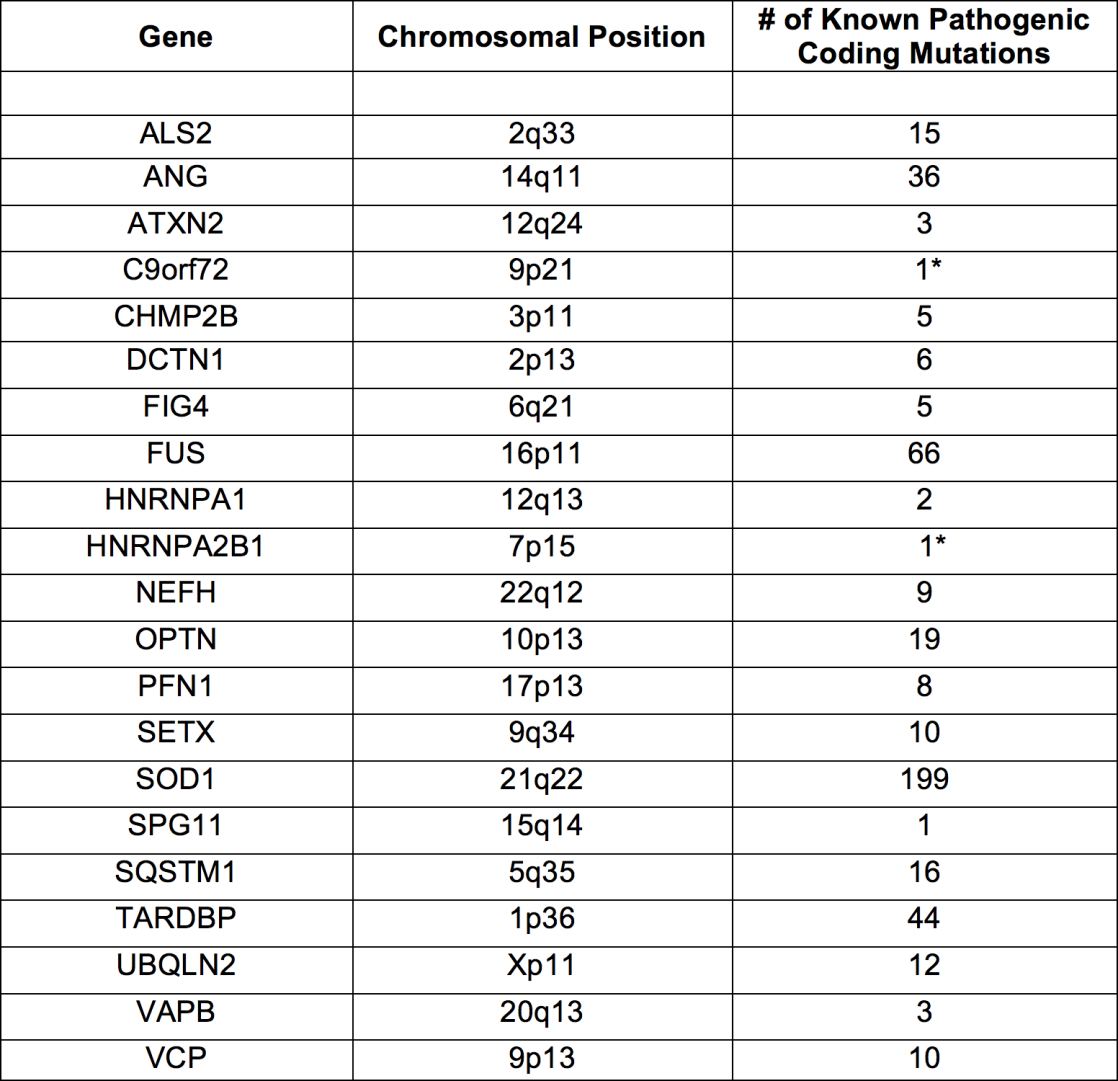
Genes pathogenic when mutated for fALS.

We used GATK [46] to identify variants genome-wide in each of our ALS samples, then extracted each sample’s variants located within the boundaries of the 21 genes in Fig 1 (S1–S7 Tables). We next assessed whether our ALS samples carried any of 471 “pathogenic” coding variants previously identified in these 21 genes (S8 Table). We compiled this list of mutations by gathering all coding (promoters, UTRs, and exons) variants classified as “pathogenic” in relation to ALS in at least one of three separate databanks (the databank http://alsod.iop.kcl.ac.uk/Statistics/pathogenicity.aspx referenced in [43], [47, 48].

Fig 1 lists genes with known causal ALS mutations, along with their chromosomal position and number of "pathogenic" ALS variants reported in at least one of three databanks queried. C9orf72 and HNRNPA2B1 only had "potentially" pathogenic variants listed, and ELP3 was excluded, as it did not have any reported mutations in these databanks.

We discovered an ALS sample (ALS4) carried a “pathogenic” variant from this list. This variant is a missense mutation (A4V) in the superoxide dismutase 1 (SOD1) locus, and was found in nearly half of ALS4’s sequencing reads (754/1576) aligning to SOD1. This likely suggests there was no transcriptional preference for the wildtype or mutant DNA sequence. No other pathogenic variants were found in ALS4 or the remaining ALS samples. None of these 21 genes were differentially expressed between our patient and control samples.

### Differential Gene Expression Testing and Associated Cellular Processes

We elected to identify ALS group-specific DEG’s using Cufflinks/Cuffdiff2, DESeq2, and EdgeR. Each tool shows strengths and weaknesses in DEG calling that vary depending on experimental conditions [49], so we ran all three to minimize biases. Samples averaged 18,750,634 paired-end reads uniquely assigned to known hg19 genes (excluding rRNA/tRNA/mtRNA genes) for EdgeR and DESeq2 analyses (S2 Fig).

**Fig 2:**
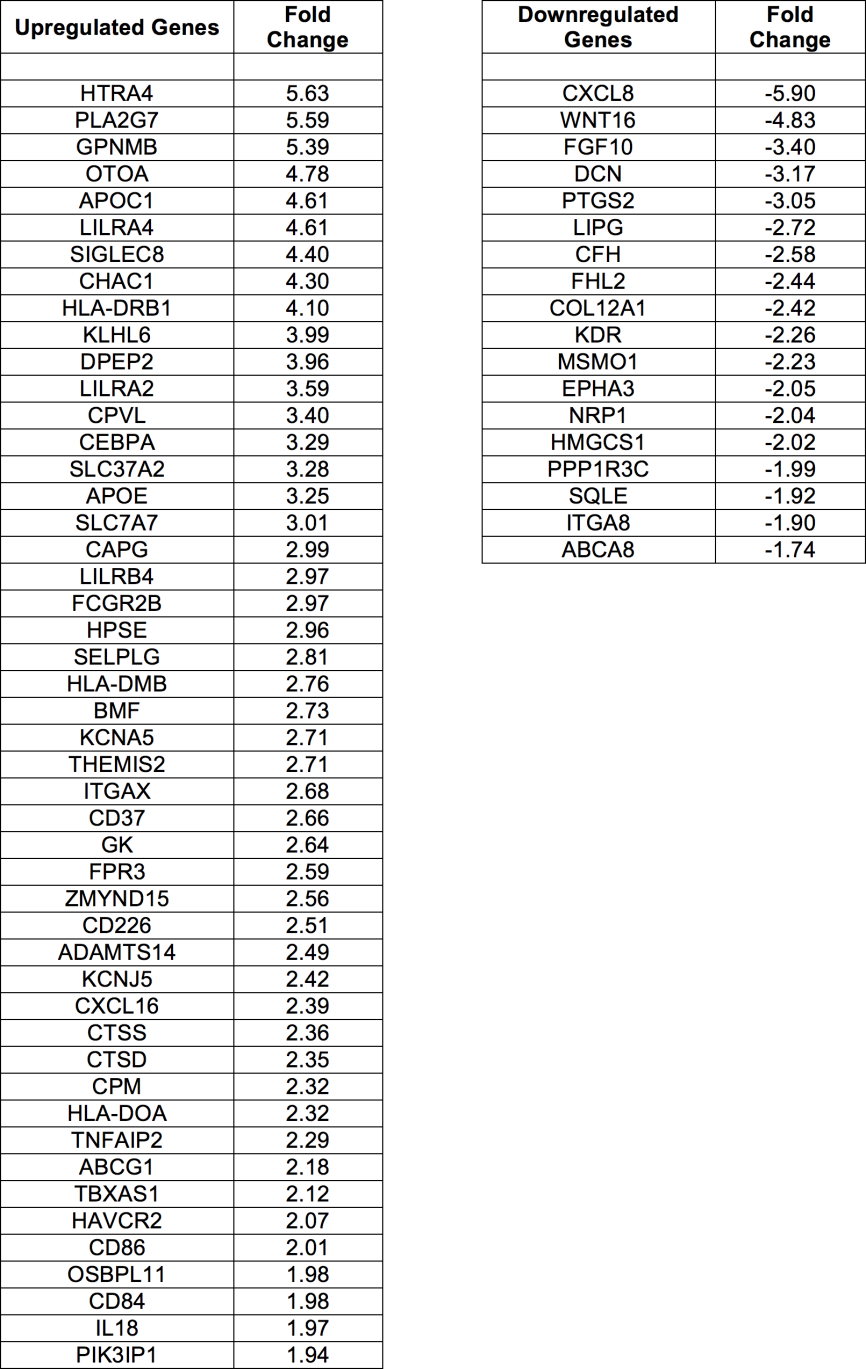
Shown are lists of common DEGs identified as “upregulated” (left) and “downregulated” (right) in the ALS group relative to controls across all three DEG analyses. Fold change values from Cuffdiff2 are listed.

At an FDR of .10, Cuffdiff2 identified 425 DEG’s, DESeq2 identified 175 DEG’s, and EdgeR identified 103 DEG’s (S9–S11 Tables). There were 74 DEG’s identified in common (56 upregulated and 18 downregulated) across all three analyses (Fig 2).

### Weighted Gene Co-Expression Analysis (WGCNA) and the “Black” Module”

**Fig 3:**
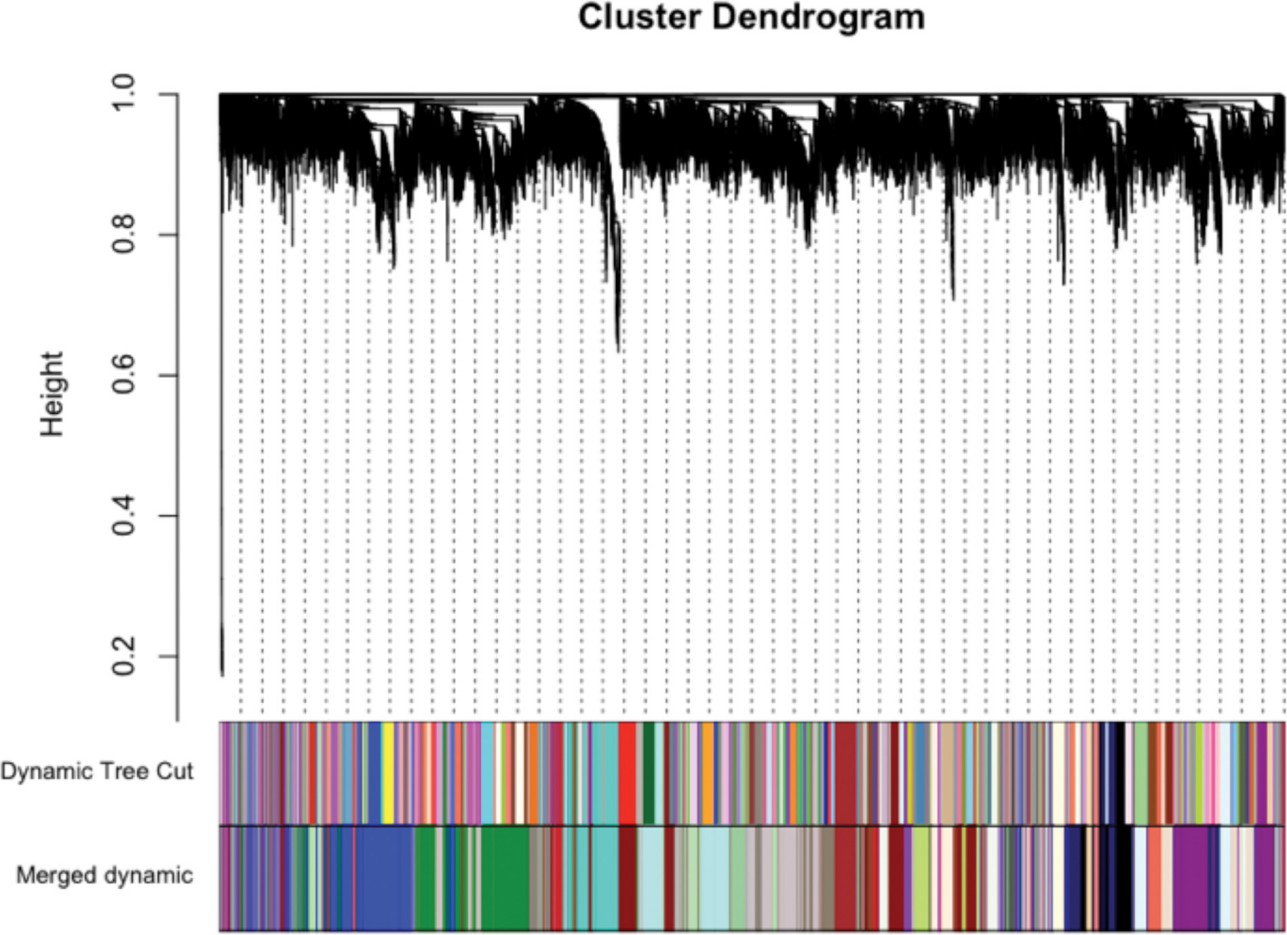
Shown are all 13,301 genes (individual black lines at top) clustered by their topological overlap dissimilarity scores. The multi-colored panel next to “Dynamic Tree Cut” shows 122 identified modules using the Dynamic Tree Cut algorithm. The second multi-colored panel shows 37 larger modules identified after highly correlated smaller modules were merged together.

**Fig 4:**
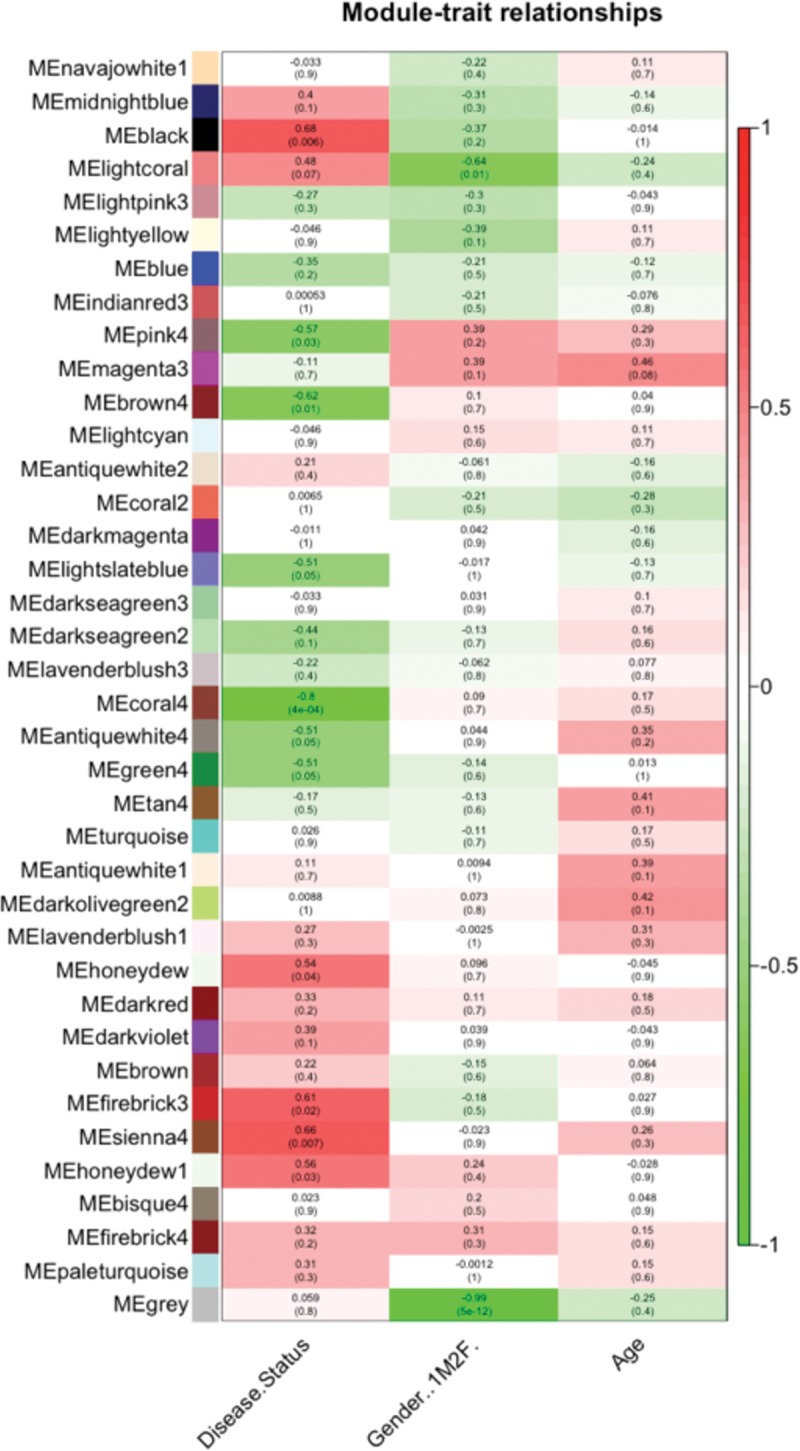
Shown are each module eigengene’s correlation values with disease status, gender, and age. The correlation score is listed above an associated p value.

We used WGCNA [42] to identify gene “modules”, or networks, from our dataset. This unsupervised technique identified 37 interconnected gene modules (arbitrarily assigned to different colors) from a filtered list of 13,301 genes (Fig 3) without using 1) information about what genes have been shown to interact in previous literature, or 2) information about which samples were cases or controls. Two of these modules (“black” and “sienna4”) were positively correlated with disease status at an uncorrected p-value < .01 (Fig 4).

### Ingenuity Pathways Analysis (IPA®)

We next used QIAGEN’s Ingenuity Pathway Analysis (IPA®, QIAGEN Redwood City, www.qiagen.com/ingenuity) to identify cellular processes and upstream regulators associated with the up- and down- regulated DEG’s identified in our ALS samples relative to controls. Downregulated DEGs were associated with various cholesterol biosynthesis and inflammatory processes (S3 Fig).

**Fig 5:**
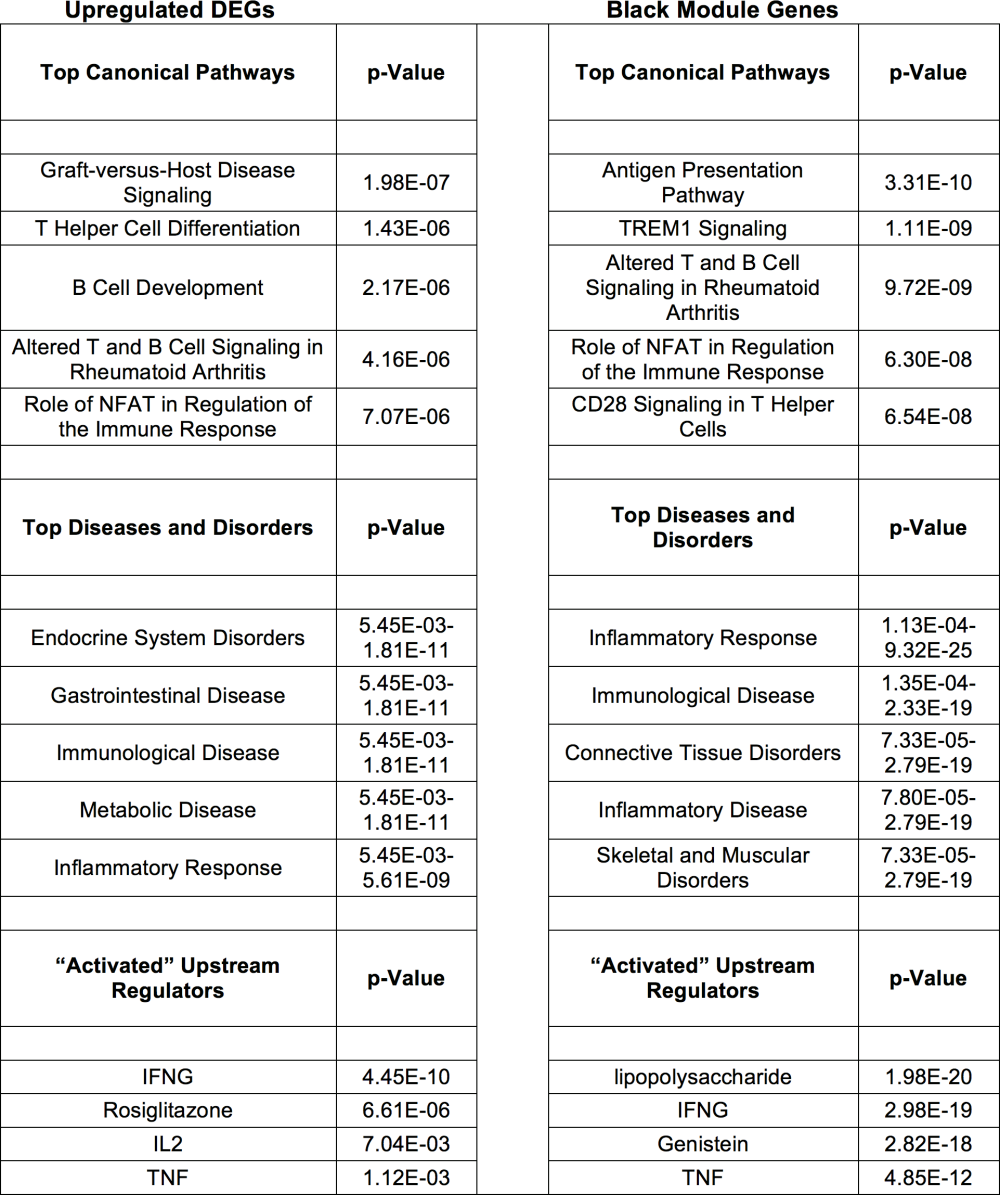
Select IPA Core Analysis Results are displayed for DEGs (56) identified as upregulated in the ALS group by all three analyses (left) and genes (495) comprising the black module (right).

Interestingly, the 56 up-regulated DEG’s were associated with multiple inflammatory processes (Fig 5), and tumor necrosis factor (TNF) was identified as a significant upstream regulator.

Interestingly, IPA revealed the 495 genes comprising the module (S12 Table) most strongly correlated to disease status (MEblack, R = 0.68, p = 0.006) were associated with inflammatory processes and TNF was a predicted upstream regulator (Fig 5). As these IPA results were highly similar to those for up-regulated DEGs in our ALS tissues, we next tested whether any of those DEG’s were found in this module. Intriguingly, we found 64% (42/65) of our up-regulated DEG’s were contained in this module (S4 Fig).

We decided to prioritize identified hub genes in this module for candidate gene selection. We found it compelling that genes in this disease-associated module and our sALS-group specific up-regulated DEG’s shared associations to inflammatory processes and up-regulated TNF signaling, despite discovery of both gene sets using independent exploratory approaches. These findings are consistent with prior proposals that inflammatory processes and TNF signaling play a role in ALS pathology [19–33].

### WGCNA Hub Gene Identification

A user can identify module hub genes using either WGCNA’s “intramodular connectivity” score or “modular membership” score calculated for every gene in a module of interest. The intramodular connectivity score reflects the cumulative connection strength a given module gene has with all other module genes. The modular membership score reflects how representative that gene’s expression values are of the module as a whole.

Hub genes typically have large values for both of these metrics. To further prioritize one hub gene over another, the authors of WGCNA [42] recommend using the “gene significance” score. This score reflects how strongly a single gene’s expression values correlate with disease status across samples.

**Fig 6:**
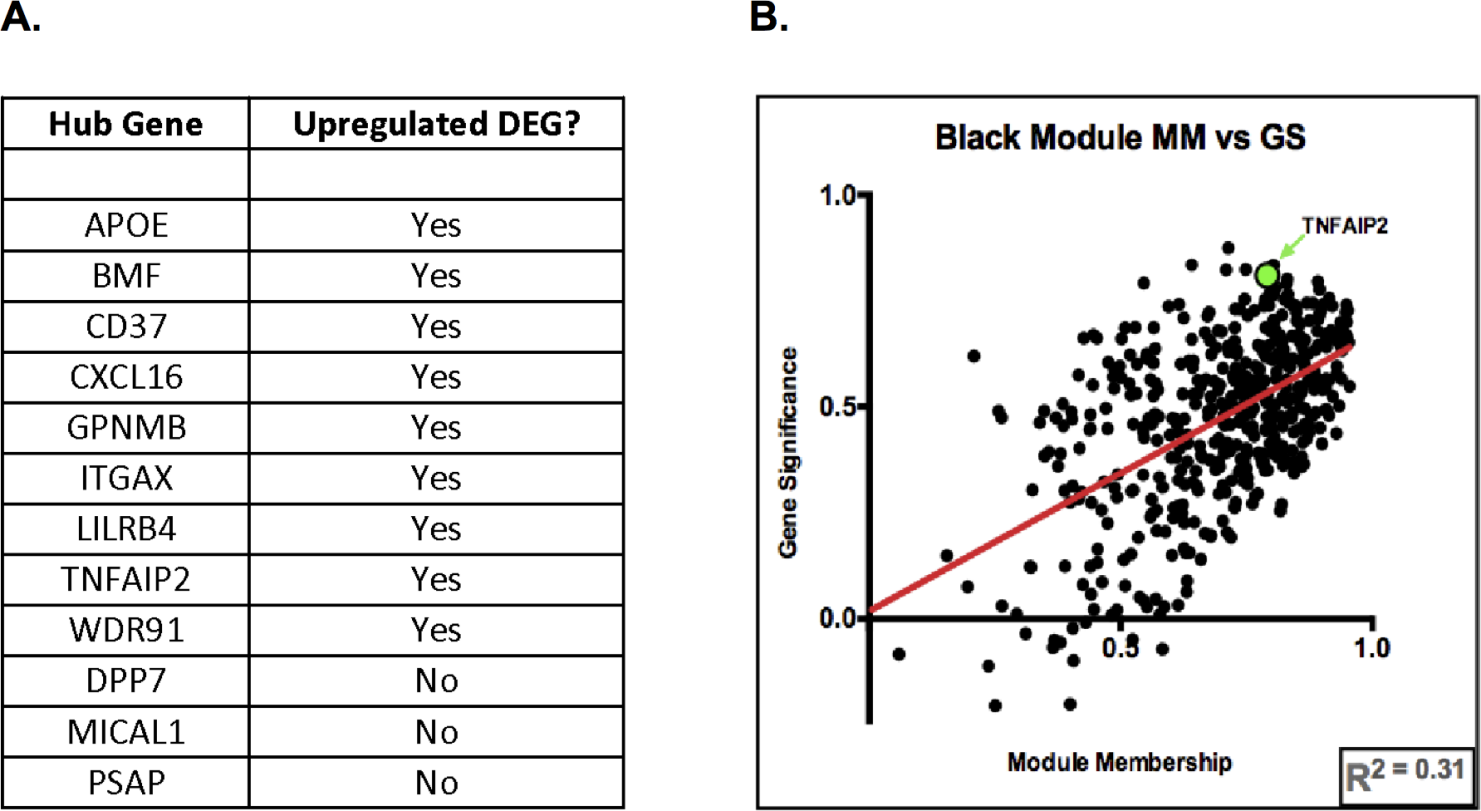
6A shows all black module hub genes and which was an upregulated DEG in the ALS group. 6B shows each black module gene’s module membership vs. gene significance scores.

12 genes in the “black” module had scores in the top quartile for intramodular connectivity, modular membership, and gene significance metrics (S12 Table). 9 of these genes were separately identified as up-regulated DEG’s in the ALS group (Fig 6A). TNFAIP2, a gene encoding an intracellular protein of the tumor necrosis factor family, was one of these nine. Fig 6B shows all “black” module genes’ modular membership vs. gene significance scores, and TNFAIP2 is highlighted in green (MM = 0.79, GS = 0.81).

We then sought to determine if the ALS hub gene/DEG group (Fig 6A) shared transcription factors that control gene expression. We input these 9 hub/DEG genes into oPOSSUM-3 [50] and after ranking by Z-score, found over-representation of TF’s mediating inflammation (REL; Z = 3.33, NFkappaB; Z = 3.08, NFkB1; Z = 3.18) and macrophage function (NR1H2::RXRA; Z = 3.33)

### Selection of TNFAIP2

We selected TNFAIP2 as our candidate gene for molecular testing as a result of data-driven findings in our study. First, TNFAIP2 belonged to the “black” module associated with ALS disease status, inflammatory processes, and TNF signaling. Second, TNFAIP2 was identified as one of twelve module hub genes with scores in the top quartile for intramodular connectivity, modular membership, and gene significance metrics. Third, TNFAIP2 was separately identified as an up-regulated DEG in our ALS samples using all three DEG-seeking algorithms.

We also considered previous research findings. TNFAIP2 is an intracellular protein component of the exocyst, and a member of the tumor necrosis factor family. TNF signaling and inflammation have long been suspected in ALS pathology [19–33]. To our knowledge, TNFAIP2’s cellular functions in motor neurons have not been studied. TNFAIP2 is known to increase in response to extracellular TNF [51–53], and has been separately associated with increased apoptosis [54–56]. We hypothesized TNFAIP2 functionally mediates motor neuron death via TNF signaling in motor neurons.

### Molecular Testing of TNFAIP2

To test our hypothesis, we assessed cell viability and activated caspase 3/7 levels in human motor neurons differentiated from induced pluripotential stem cells [57–59] that either 1) overexpressed a plasmid encoding TNFAIP2 tagged with an C-terminal GFP (S5 Fig) or 2) overexpressed the same plasmid encoding GFP alone (empty vector).

These motor neurons had a ~15–22 fold increase in expression of motor neuron specific markers HB9 and ISL1 at day 21, suggesting successful differentiation (58). qPCR data (S13 Table) showed TNFAIP2 expression increased >300-fold in motor neurons transfected with TNFAIP2-GFP relative to GFP alone.

**Fig 7:**
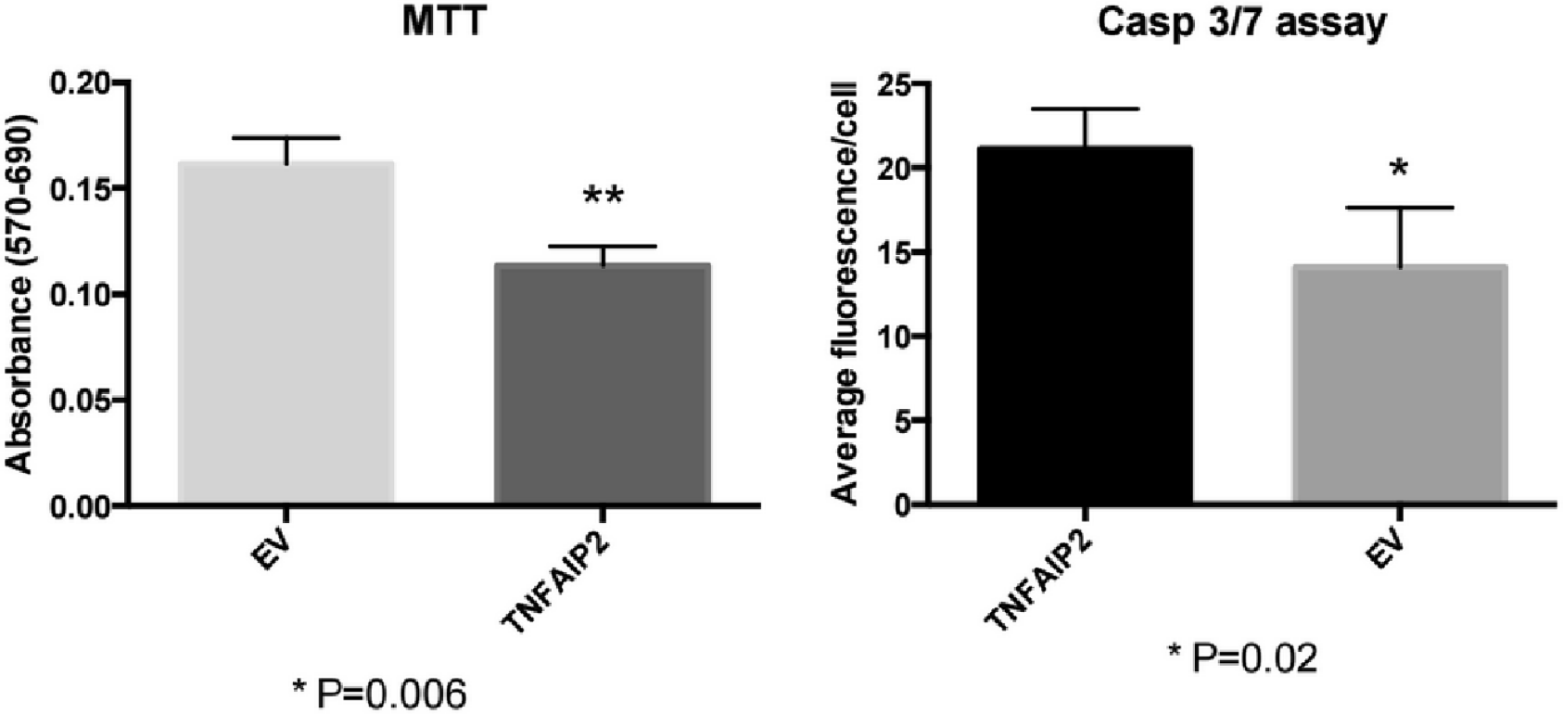
(left, “MTT”) shows absorbance readings reflecting MTT metabolism (a function viable cells perform) On day 21 of differentiation cells were transfected with TNFAIP2-GFP (TNFAIP2) vector or GFP alone vector (EV) and MTT cell viability assay was performed 24 hrs later. Activated caspase 3/7 levels (right, “Casp 3/7 assay”) were measured using fluorescently labeled antibodies. Day 21 motor neurons that overexpressed TNFAIP2-GFP (TNFAIP2) compared to GFP alone (EV) were significantly less viable and have significantly more caspase 3/7 staining 24 hours after introduction of the TNFAIP2 vector. Statistical analyses were carried out using unpaired t-tests and Prism® software.

The MTT cell viability assay showed motor neurons that overexpressed TNFAIP2-GFP were significantly less viable, and a caspase 3/7 assay revealed these motor neurons had significantly increased levels of activated caspase 3/7 compared to motor neurons that overexpressed GFP alone (Fig 7). Taken together, these results suggest overexpression of TNFAIP2 results in motor neuron death, and may play a functional role in ALS pathology.

## Discussion

sALS is a complex trait without a viable treatment, and its polygenic contributions remain poorly understood. In this study, we combined deep RNA-sequencing, systems biology analyses, and molecular biology assays to elucidate ALS group-specific differences in postmortem spinal tissue that may be relevant to disease pathology. To our knowledge, this is the only study that exploits the benefits of next generation RNA-Sequencing [60, 61] to measure gene expression changes in postmortem cervical spinal tissue containing disease-vulnerable motor neurons from sALS patients. We chose to study gene expression changes in human postmortem sALS tissue over fALS rodent tissue, as recent findings suggest the majority of sALS cases do not carry a known monogenic causal mutation [1] and this tissue is inaccessible at earlier stages of the disease. The only other RNA-Sequencing study in human postmortem sALS tissues we are aware of used cerebellar and prefrontal cortex tissues [62], and also found sALS-group specific DEGs were associated with inflammatory processes.

Previous ALS studies have used gene network analyses to unveil disease-associated cellular processes. Those using gene co-expression network analyses identified ALS tissue networks associated with innate immune response, stress response, post-translational modifications, inflammatory disease, and neurological disorders [63, 64]. Other ALS studies used gene network analyses that model gene-gene interactions using different criteria. These criteria include only connecting genes with known interactions in previous literature, or using information theoretics to eliminate indirect connections between genes inferred to be connected based on the strength of their co-expression value alone. These studies identified ALS-specific networks associated with organismal injury, immune response, post-translational modification, regulation of the actin cytoskeleton, and extracellular matrix repair [65, 66]. Ikiz et al applied the MARINa algorithm to identify major regulators (such as transcription factors) within a gene network identified in motor neurons in an *in vitro* ALS model characterized by MN death [67]. They identified 8 genes as drivers of motor neuron death including NFKB1, a pleiotropic transcription factor with important roles in innate immune response. Taken together, these findings support the use of systems-level gene network analyses to identify perturbed cellular processes in ALS tissues, and hold potential to unveil therapeutic target genes.

In this study, we discovered only one of our ALS samples carried a known causal ALS mutation out of 471 mutations surveyed. We found inflammatory processes and TNF signaling were significantly associated with sALS group-specific gene expression changes (IPA) using independent exploratory DEG tests (Cuffdiff2, DESeq2, and EdgeR) and an unsupervised gene network analysis (WGCNA). Finally, we demonstrated overexpression of the hub gene TNFAIP2 in motor neurons differentiated from human iPSCs *in vitro* led to decreased cell viability and elevated levels of caspase 3/7, findings suggestive of increased cell death. Taken together, these results corroborate previous reports that most instances of sALS have polygenic contributions [1], and inflammatory TNF signaling plays an important role in sALS pathology [19–33].

Modulating TNF signaling activity may be effective in slowing disease progression. TNF is a potent inflammatory cytokine that plays an instrumental role in cell fate decisions. TNF-mediated pro-survival processes are largely effected via upregulation of the transcription factors NFKB1 and JUN [68], whereas its cell death processes are ultimately carried out by initiator and effector caspases. Bioactive forms of TNF commence these processes via two cell surface receptors, TNFR1 and TNFR2. TNFR1 directs cell survival or death, whereas TNFR2 is only known to promote pro-survival effects [34]. The extracellular domains of TNFR1 and TNFR2 are shed into general circulation after interacting with bioactive forms of TNF, and function in a negative feedback loop as they retain their ability to bind TNF [69]. Intriguingly, elevated levels of TNF-alpha and extracellular domains of TNFR1 and TNFR2 have been found in the blood [28] and serum [29, 30] of human ALS patients compared to controls.

We propose novel therapies to reduce TNF synthesis in human sALS patients, monitoring blood levels of TNF and these extracellular TNFR domains as biomarkers throughout treatment. Two potential therapeutic agents are bupropion and curcumin. Bupropion, a drug commonly used to treat clinical depression, decreased TNF serum levels in mice likely via increasing intracellular cAMP signaling after binding beta-adrenergic and/or D1 receptors [70]. Curcumin, an anti-inflammatory compound in turmeric, reduced TNF transcription in human cancer cells [71, 72] and lipopolysachharide (LPS)-stimulated murine microglia [73]. This likely occurs via inhibition of NFKB1, a transcription factor that is upregulated by TNF signaling and known to induce TNF and other inflammatory cytokines [74]. Curcumin is also predicted to bind and inhibit caspase-3 [75], an activator caspase with a known role in TNF-mediated apoptosis. Curcumin oral bioavailability and brain penetration were substantially increased by micellular formulation [76], setting the stage for clinical testing of it and bupropion separately and together to reduce TNFα signaling.

Thalidomide, a TNF synthesis inhibitor, has been reported in a Phase II trial to not alter ALS progression[77]. However, the authors were unable with thalidomide to alter serum levels of any cytokine, including TNF (see their Fig 6). These results suggest that the thalidomide doses used, which were associated with side effects in some subjects, did not reduce TNF synthesis.

Although we focused on TNFα signaling and modulated the hub gene TNFAIP2 in an ALS-vulnerable cell type (human motor neurons) in our analyses, we do not claim aberrant inflammatory TNFα signaling as the sole factor in ALS pathogenesis. Downregulated DEGs in the ALS group and genes in the other disease-associated module were associated with other cellular processes (S3 and S6 Figs).

We also identified 8 hub genes that were upregulated DEGs within the “black” module that was identified by WGCNA and was statistically associated with ALS. These genes are involved in different gene families and could serve as foci for additional mechanistic studies and therapeutic interventions.

Genetic variants in *APOE* (Apoliprotein E), a gene encoding a protein important for transporting cholesterol and other lipids between cells, alter its function and are associated with an accumulation of amyloid-Β peptides in the brain and an increased Alzheimer’s risk. These variants modify ALS age of onset and features of disease progression [78], warranting investigation into whether aberrant expression of APOE could also influence ALS pathology.

*BMF* (Bcl2-modifying factor) binds to Bcl2 and related anti-apoptotic proteins and promotes apoptosis [79, 80]. Its expression is increased in human motor neurons exposed to TNFα protein (data not shown), and could play an important role in motor neuron death.

*CD37*, a leukocyte-specific protein belonging to the tetraspanin superfamily, is integral to T cell proliferation [81]. Aberrant expression of this gene may influence T cell activities during innate immune responses implicated in ALS.

*CXCL16* (Chemokine ligand 16), a transmembrane chemokine produced by reactive astroglial cells, is increased by TNFα [82] and induced by neurodegeneration. It promotes CXCR6-positive glial cell invasion that favors astrogliosis [83], a feature seen in ALS CNS tissues.

*GPNMB* (Glycoprotein NMB) was previously identified as an upregulated DEG in the spinal cords of fALS rodents. Interestingly, extracellular fragments of GPNMB released by activated astrocytes lessened the neurotoxicity of mutant SOD1, suggesting it may play protective role against neurodegeneration [84].

*ITGAX* (Integrin Alpha X), a leukocyte-specific integrin, was found as an upregulated DEG in leukocytes that invaded the spinal cords of fALS rodents at different stages of disease progression [85]. ITGAX plays a known role in cell-cell interactions during immune responses, warranting further research into its therapeutic potential in halting such processes that precede neurodegeneration.

*LILRB4* (Leukocyte Immunoglobulin-Like Receptor Subfamily B Member 4), a cell surface receptor in immune cells, binds MHC class 1 molecules to inhibit immune responses. While not directly studied in ALS tissues, LILRB4 expression negatively correlates with pathologic inflammation in a mouse model of allergic pulmonary inflammation [86].

To our knowledge, no study has investigated the function of *WDR91* (WD Repeat Domain 91), so it is impossible to speculate on its possible connection to ALS pathology.

Our study has several important limitations:

First, we examined a small number of postmortem cervical spinal cord sections for gene expression. There are ~30,000 persons with ALS in the US. The cost of RNA-seq analysis limited the numbers of cases we could examine at the sequencing depth employed. As a result, it is impossible to state to what degree our findings can be generalized to thousands of patients.

Second, we used postmortem tissue. As a result, we are examining gene expression of cells (mainly astrocytes) that are “survivors” of the neurodegenerative process. To what extent ALS modifies cellular gene expression over time is not known, and it is not possible presently to examine human CNS tissues across disease progression. It is unclear whether the “young” motor or other neurons produced by iPSC approaches will approximate changes seen in spinal motorneurons present for many years as ALS progresses.

Third, RNA-Seq aligners have built-in biases that influence results in different ways, and our alignments are subject to those conferred by Tophat2 and STAR (for the point mutation analyses). Engstrom et al [87] compared alignment results from 26 mapping protocols on 4 common RNA-Seq datasets. Tophat2 reported a smaller number of alignments due to its low tolerance for mismatches, but a higher number of splice sites when used with a guide annotation. STAR reported a larger number of primary alignments as it retains portions of a read when unable to align the entire sequence, but also a greater number of false exon junctions. It remains unclear which tools are best suited for different downstream analyses. As RNA-seq technology continues to mature, future algorithms should improve in terms of these biases and it will become more clear which tools are best to use for a given purpose.

Fourth, we did not yet explore novel transcripts, indels, or selective exon usage specific to our sALS group in this dataset. We also wish to investigate expression of smaller non-coding RNA’s, particularly microRNA’s, that regulate mRNA stability in a future study. We did find substantial presence of mtDNA gene deletions with variable deletion burdens across mtDNA-encoded respiratory genes (data not shown) that we will present in a subsequent paper.

We anticipate future exploratory sALS studies will continue to uncover polygenic contributions and identify potential therapeutic targets. Genome-wide Association Studies (GWAS) comparing sALS cases vs. neurologically healthy controls were instrumental in the discovery of excess pathogenic non-coding repeats in C9orf72 found in 7% of Caucasian sALS patients [1]. Chesi et al [5] identified excess de novo mutations in chromatin regulator genes using exome sequencing, comparing sALS offspring with their neurologically healthy parents. This ALS gene expression study joins those preceding it in identifying perturbed cellular processes and corroborating them using separate molecular biology assays [1] [19–33]. Recent findings suggest considerable clinical heterogeneity between sALS patients [3, 88]. As sequencing costs decrease, larger sample sizes conferring greater statistical detection power will become feasible. These data sets will likely enable stratification of sALS by its varied molecular phenotypes as has been seen in other diseases like breast cancer [89]. These approaches may ultimately lead to therapies against pathways that are universally beneficial to sALS patients, such as TNF signaling, as well as those specifically tailored to an individual patient’s pathophysiology.

## Materials and Methods

### Sample Demographics

Each sample’s age, ethnicity, and gender information can be found in S7 Fig. ALS Samples: 67.71 +/- 7.99 years of age, 7 Caucasian, 4 Male 3 Female. Control Samples: 69.75 +/- 11.29 years of age, 6 Caucasian 2 African American, 4 Male 4 Female.

### Sample Preparation and Sequencing

Frozen cervical spinal cord specimens from all 15 samples were procured via the National Disease Research Interchange, Philadelphia, PA (http://www.ndri-resource.org) and stored at -80 degrees prior to usage. All samples were provided anonymously and were de-identified and coded by number only. Total RNA was extracted from twenty 20-μm frozen cross sections cut (at -20 degrees) using a Cryostat. We used the miRNeasy Mini (Qiagen) RNA extraction kit, including the optional on-column DNAse step to prevent reads derived from DNA in downstream analysis. We further purified this RNA using the Qiagen RNeasy Micro kit to remove organic contaminants.

RNA for all samples was quantified using a Nanodrop 2000c spectrophotometer (Thermo Scientific) and quality was assessed using the Experion® automated electrophoresis system (Bio-Rad). Bio-Rad’s Experion® calculated an RNA Quality Index (RQI) score via comparing three portions of a sample’s electrophoretic profile to a manufactured standard of degraded RNAs. RNA Quality Index (RQI) values range between 1–10, with increasing values representing higher quality RNA. 500 ng of RNA from each sample with an RQI score ≥7 was used for library construction.

The Illumina Truseq Stranded Total RNA HT Sample Prep Kit ® instructions were followed to generate barcoded RNA-Sequencing libraries for all eligible samples. We confirmed libraries had the expected sized band (~260 bp) using the Experion automated electrophoresis system (Bio-Rad). We then quantified libraries using the KAPA library quantification kit (Kapa Biosystems, Wilmington, MA). Barcoded RNA-Sequencing libraries were equimolar pooled and added to the Illumina Nextseq 500 lane for multiplex sequencing at Cofactor Genomics (Saint Louis, MO). Data was processed using the standard Illumina processing pipeline to segregate each multiplexed sample’s reads, and raw fastq files for each sample were sent to us for further processing.

### Data Pre-Processing and Alignment with Tophat

FastQ files for each individual sample were input to FastQC (http://www.bioinformatics.babraham.ac.uk/projects/fastqc/) for quality assessments. This software identifies any data quality issues across metrics including: base quality per position across reads, overall GC content, sequence length distribution, and duplicate read frequency. All samples passed this QC check (data not shown).

Samples were next processed using Trimmomatic [90]. Trimmomatic removed all Illumina adaptor sequences and bases with a Phred quality score less than 20 from the 3’ end of our reads. A Phred score of 20 indicates a 99% probability the base is correctly identified. Fragments > or = to 50 bp were retained after that step. We took these filtering steps to ensure high quality reads were used for alignment, as the average PHRED score in bases towards the 3’ end of reads across samples decreased (data not shown).

We next used the Burrows-Wheeler Aligner (BWA, [91]) to calculate insert size metrics for each sample (including average size and standard deviation) to improve Tophat2 alignment. Once these metrics were obtained, we aligned each sample’s reads to the hg19 human reference transcriptome then genome using Tophat2 [92]. The hg19 reference transcriptome and genome were derived from Illumina iGenomes UCSC hg19 directory downloaded from the Tophat webpage (https://ccb.jhu.edu/software/tophat/igenomes.shtml). Examples for these commands can be found in S1 Note.

### Calculation of Sequencing Alignment Metrics

We used Picard Tool’s (http://picard.sourceforge.net) CollectRNASeqMetrics command to gather alignment data for each sample. We input each sample’s Tophat2 aligned reads file and the “refFlat.txt.gz” file provided in the aforementioned Illumina iGenomes directory into each command. We created a “ribosomal intervals file” containing rRNA, tRNA, and mitochondrial RNA species as we wished to know how many reads aligned to these regions collectively. An example of this command can be found in S1 Note.

### Identification of sequencing variants in each sample

We downloaded STAR [93] and used it along with other necessary software (GATK, PicardTools) to complete the GATK pipeline (https://www.broadinstitute.org/gatk/guide/article?id=3891) for identifying sequencing variants using RNA-Sequencing reads. The genome.fa file from the aforementioned Illumina iGenomes directory was used along with each sample’s Trimmomatic-processed fastq files for STAR alignment. We bypassed the optional indel realignment step, and used default settings for all steps. We downloaded the dbsnp_138.hg19.vcf (variant call file) for use in several commands from ftp://ftp.broadinstitute.org/bundle/2.8/hg19/, as detailed on this GATK webpage (https://www.broadinstitute.org/gatk/guide/article?id=1215).

We created a file containing all 21 fALS genes’ exon boundaries from the genes.gtf file in the aforementioned Illumina iGenomes directory. We then excluded each sample’s variants discovered outside of these exon boundaries. We next checked whether any of the remaining variants corresponded to one of the “pathogenic” ALS mutations catalogued in at least one of three databases queried (the databank http://alsod.iop.kcl.ac.uk/Statistics/pathogenicity.aspx referenced in [43, 47, 48]).

An example of the GATK pipeline commands used for each sample can be found in S1 Note.

### HTSeq count to produce individual sample count matrices for DeSeq2 and EdgeR

We next generated individual sample count matrices using HTSeq-Count [94] that reported the number of aligned read pairs that uniquely map to known genes in the hg19 genome. We used our Tophat2 mapped reads and the hg19 genes.gtf file provided in the aforementioned Illumina igenomes directory to accomplish this. The counting procedure this software uses is described on their website (http://www-huber.embl.de/users/anders/HTSeq/doc/count.html).

An example command for each sample can be found in S1 Note.

### Cufflinks/Cuffdiff DEG Analysis

Gene and transcript abundances for all samples were estimated using Cufflinks v2.2.1 (http://cufflinks.cbcb.umd.edu/howitworks.html, [39]). We opted to mask all rRNA, tRNA, and mitochondrial RNA mapped reads from FPKM calculations, as these RNA species accounted for different proportions of total mapped reads in each sample (S1 Fig). We only considered reads aligning to known genes (compatible hits norm flag) in our FPKM calculations, as we suspected the number of novel transcripts varies across samples. We also used the genome bias and multi-hits correction flags, as recommended by the Tuxedo Suite developers.

We used Cuffmerge to merge all samples’ transcripts.gtf files prior to running Cuffdiff2. We then ran Cuffdiff2 to test for differential expression of known genes in the hg19 genome. Again, we opted to use the genome bias and multi hits correction flags, and masked out all rRNA, tRNA, and mitochondrial RNA mapped reads from for differential testing.

We calculated Benjamini-Hochberg corrected p-values using input Cuffdiff reported p-values for each gene via the R function p.adjust. We considered DEGs significant if their Benjamini-Hochberg corrected p-value was < 0.10. Examples of these commands are in S1 Note.

### DESeq2 DEG Analysis

HTSeq count files for each sample were placed into a created DESeq2 directory prior to DESeq2 [40] analysis in R.

We performed the DEG analysis using input HTSeq count matrices as prescribed in the DESeq2 vignette. We considered DEG’s significant if their adjusted p values were < .10. These commands are included in S1 Note.

### EdgeR DEG Analysis

HTSeq count files for each sample were placed into a created EdgeR directory prior to EdgeR [41] analysis in R. We then created a file containing all samples’ count information for all genes prior to following the prescribed EdgeR analysis. We decided to filter out genes with a cpm (counts per million) value <1 in 7 samples. We chose a cpm value of 1 as smaller values more likely reflect noise. We chose 7 for our sample threshold as genes that were only expressed in the disease or control group could play an important role in disease pathology.

We calculated Benjamini-Hochberg corrected p-values using EdgeR reported p-values for each gene via the R function p.adjust. We considered DEG’s significant if their Benjamini-Hochberg corrected p-value was < 0.10. Examples of these commands are in S1 Note.

### Weighted Gene Co-Expression Network Identification and Association Testing

WGCNA [42] follows a 6-step process to predict which genes are connected to each other, cluster them into gene networks, test which gene networks are associated with disease status, and aid user selection of hub genes. The mathematical formulas used in each step are not included in this description, but can be found in an earlier publication [95].

First, WGCNA calculates an “adjacency matrix” (a gene network) that reports a correlation value between every pair of genes’ expression measurements across all 15 samples. An underlying assumption is the higher the correlation value between a pair of genes, the more likely it is they are functionally connected. Once the adjacency matrix is constructed, summation of any individual gene’s correlation values to all other genes reflects its level of overall connectedness.

Second, the adjacency matrix is raised to a software-determined exponential power, thereby reducing noise by pushing weaker pairwise connection values closer to zero relative to stronger values. The exponential power used is the lowest value needed to ensure the network approximates scale-free topology. In this context, scale-free topology is satisfied when a small number of genes (hub genes) are highly connected to other genes, whereas the majority of genes are weakly connected to other genes. Many biological (including gene co-expression) networks demonstrate scale-free topology [95], and their network functions are more likely disrupted by specifically targeting highly connected members [96, 97]. This step lays the foundation for identification of hub genes within smaller modules (networks) of interest later in this analysis.

Third, the adjacency matrix is transformed into a “topological overlap” matrix by calculating topological overlap (TOM) scores for each gene. This score accounts for each pair of genes’ connection strength (adjacency value) to each other as well as their connection strengths (adjacency values) to every other gene in the adjacency matrix. Higher TOM scores indicate a pair of genes is more likely connected to each other and a shared set of other genes.

Fourth, WGCNA identifies gene co-expression networks via average linkage hierarchical clustering using a TOM-based dissimilarity measure (1-TOM score for every gene) as a measure of distance. The resultant dendrogram of clustered genes is segregated into individual modules with at least 30 genes using WGCNA’s dynamic tree-cutting algorithm [42].

Fifth, WGCNA calculates each module’s “eigengene”, or first principle component, using all samples’ gene expression values for all genes in that module. A module eigengene is considered a summarized expression profile representative of that module for all samples. Each module eigengene is then correlated against every other module eigengene. If two or more modules’ eigengenes have a correlation value >.75, those modules are merged together generating a larger module. Module eigengenes are re-calculated at this stage and the process is repeated until there are no module eigengenes that correlate to each other with a value >.75.

Finally, each module eigengene is tested for statistical association to user-provided continuous or binary phenotypic traits, including disease status. Correlation test p-values based on the student test are reported, and are equivalent to a Wald test in a linear regression.

### Weighted Gene Co-Expression Network Analysis

We first generated a filtered list of 13,301 genes and their Cufflinks FPKM values for all samples to analyze in WGCNA. All of these genes had an FPKM value >2 in at least 7 samples. We chose an FPKM of 2 as smaller values more likely reflect noise. We chose 7 for our sample threshold as genes that are only expressed in the disease or control group could play an important role in disease pathology. We log-transformed these FPKM values using log2 (FPKM value +1) as recommended on the WGCNA FAQ’s page (http://labs.genetics.ucla.edu/horvath/CoexpressionNetwork/Rpackages/WGCNA/faq.html) prior to analysis.

Our analysis was guided by steps 1, 2b, and 3 in the R Tutorial listed under “I. Network analysis of liver expression data from female mice: finding modules related to body weight” from their website (http://labs.genetics.ucla.edu/horvath/CoexpressionNetwork/Rpackages/WGCNA/Tutorials/).

We deviated from the tutorial several times. We generated a “signed weighted” adjacency matrix for downstream analyses as opposed to the default “unsigned weighted” network. We chose this option to preserve the direction of a pair of genes’ correlation, as a positive correlation may indicate “activation” whereas a negative correlation may indicate “repression”. The unsigned networks do not preserve the direction of correlation. We used the “bicor” (biweight midcorrelation) correlation in place of the pearson correlation to construct our adjacency matrix and determine the exponential value necessary to approximate scale-free topology (S8 Fig).

We chose this option as we had a small sample size, and biweight midcorrelations are more robust to outliers compared to pearson correlations [42]. We added the flag “corOptions = list(maxPOutliers = 0.1))” to further reduce outlier effects.

Commands used after loading the sample data expression matrix (S14 Table) and phenotypic data (S15 Table) as instructed in part 1 of the tutorial are detailed in S1 Note.

### iPSC generation and Neural Induction

Integration-free iPSCs were generated from peripheral blood mononuclear cells (MNC) from a healthy male donor, aged 62, using a previously described protocol [57], with modifications [59]. Briefly, the pEB-C5 and pEB-Tg plasmids (Addgene) were electroporated into cells using an Amaxa Nucleofector 4D system (Lonza, Allendale, NJ). After three weeks, viable colonies were expanded in mTeSR medium on Geltrex (Life Technologies) coated plates. Neuralization of iPSCs was accomplished using PSC Neural Induction Medium (Life Technologies) according to the protocol with modifications [58]. All cultures were maintained at 37°C in a humidified CO2 incubator with the oxygen level held at 5%.

### Motor Neuron Differentiation

After neural induction, iPSCs were differentiated as described previously [58], with some modifications [59]. Briefly, adherent cells were grown in neural induction media containing DMEM/F12 with 0.2 μM LDN-193189 (LDN; Stemgent), 10 μM SB431542 (SB; Stemgent), 10 ng/ml BDNF (R&D systems), 0.4 ug/ml L-ascorbic acid (Sigma), 2 mM GlutaMAX-I supplement, 1% N-2 supplement, and 1% nonessential amino acids (NEAA). Two days later 1 μM RA was added. On day four LDN/SB was stopped and 1 μM smoothened agonist (SAG; Calbiochem or Santa Cruz) and 0.5 μM PM were added. On day 14 cells were switched to neurobasal media containing 2 mM GlutaMAX-I, 2% B-27, 1% NEAA, 0.4 ug/ml AA, 10 ng/ml GDNF (R&D), 10 ng/ml CNTF (R&D). Media was replaced every 2–3 days. Unless otherwise specified, all cell culture materials were purchased from Life Technologies. All cultures were grown at 37°C in 5% oxygen and 5% CO_2_ conditions.

### MTT/Caspase 3/7 Assays

On day 21 of differentiation cells were transfected with TNFAIP2 plasmid or empty vector control (Origene) using FuGENE HD Transfection Reagent (Promega). For transfection, 100 ng plasmid DNA was added per well of a 96-well plate and the Fugene:DNA ratio was 4:1. After 24 hours of treatment, cell viability was measured using the In Vitro Toxicology Assay Kit, MTT based (Sigma, TOX1) according to manufacturer instructions. For detection of activated caspase 3/7, the FLICA® 660 Caspase 3/7 Assay Kit (ImmunoChemistry Technologies) was used according to manufacturer instructions. For quantification, cells were fixed and 5–10 representative fields were taken with an Olympus FV1000 confocal microscope. Images were analyzed using MetaMorph image analysis software (Molecular Devices) and pixel intensity was normalized to the number of cells per image, identified by DAPI nuclear staining.

### Real-time quantitative PCR (qPCR)

For qPCR analysis, RNA was extracted with the RNeasy Plus Micro Kit (Qiagen) according to manufacturer instructions. Quantification of isolated RNA was performed using a Nanodrop 2000c spectrophotometer (Thermo Scientific). RNA was reverse transcribed into cDNA using the iScript cDNA synthesis kit (BioRad). For qPCR, 50 ng cDNA per well was loaded into a 96-well plate and analyzed with the CFX96 Real Time PCR Detection System (BioRad). All samples were analyzed in triplicate. Data was normalized to the geometric mean of two reference genes determined to have the greatest stability using the software qbasePLUS-GeNorm (BioGazelle; 14.3.3.Z and CYC1). Primer sets are available upon request. Statistics were calculated using unpaired t-test in Prism software (GraphPad, Prism). Error bars represent standard error of the mean.

## Supporting Information

S1 Fig Sample metrics collected using Picard’s CollectRNASeqMetrics command. PF = Passed Filter.
(PDF)

S2 Fig Shown are the number of paired end reads that aligned either uniquely or multiply to hg19 genes. Uniquely aligned reads were used for DeSeq2 and EdgeR analyses.
(PDF)

S3 Fig Shown are IPA Core Analysis Results for DEGs (18) identified as downregulated in the ALS group by all three analyses. (PDF)

S4 Fig Shown are DEGs identified as “upregulated” in the ALS group that were also found in the black module. Fold change values from Cuffdiff2 are listed.
(PDF)

S5 Fig Shown is the TNFAIP2-GFP plasmid used in this experiment. The empty vector used for the EV group was the same plasmid with TNFAIP2 CDS taken out.
(PDF)

S6 Fig Shown are IPA Core Analysis Results for the 67 genes comprising the sienna4 module defined by the WGCNA analysis. (PDF)

S7 Fig Individual ages, genders and ethnicities for the subjects from whom the cervical spinal cord samples used in this study were obtained. ALS = amyotrophic lateral sclerosis; CTL = control.
(PDF)

S8 Fig Shown are the effects on Scale Independence and Mean Connectivity of raising the adjacency matrix to the power of 24. The results approximate scale free topology at .80 correlation. Data are derived from the WGCNA analyses.
(PDF)

S1 Note Pre-Processing and Alignment Commands (Unix). (PDF)

S1 Table GATK analysis of tissue sample ALS1. (XLS)

S2 Table GATK analysis of tissue sample ALS2. (XLS)

S3 Table GATK analysis of tissue sample ALS3. (XLS)

S4 Table GATK analysis of tissue sample ALS4. (XLS)

S5 Table GATK analysis of tissue sample ALS9. (XLS)

S6 Table GATK analysis of tissue sample ALS10. (XLS)

S7 Table GATK analysis of tissue sample ALS14. (XLS)

S8 Table Pathogenic coding variants known in ALS. (XLSX)

S9 Table DEG's determined by Cuffdiff algorithm. (XLS)

S10 Table DEG's determined by DESeq2 algorithm. (XLS)

S11 Table DEG's determined by EdgeR algorithm. (XLS)

S12 Table "Black module" genes and their properties as determined by WGCNA. (XLSX)

S13 Table qPCR results for TNFAIP2 expression in both motor neuron groups. (XLSX)

S14 Table All Samples' log-transformed FPKM values for select genes. (XLS)

S15 Table Sample Phenotypic Data for WGCNA association testing. (XLS)
