# Supplementary material for: RNAseq Analyses Identify Tumor Necrosis Factor-Mediated Inflammation as a Major Abnormality in ALS Spinal Cord
Source: PLoS One. 2016 Aug 3;11(8):e0160520. doi: 10.1371/journal.pone.0160520 (PMC4972368; doi:10.1371/journal.pone.0160520)
Supplement: S1 Fig — PF = Passed Filter. (PDF) [file pone.0160520.s001.pdf]

Supplementary Figure 1

	Bases Sequenced	PF Bases Aligned to hg19 genome	% Bases that PF and Aligned to hg19 genome	% PF Bases aligned to mRNA	% PF Bases aligned to rRNA/tRNA/mtRNA	% PF Bases aligned to intronic regions	% PF Bases aligned to intergenic regions
**Sample ID**							
**ALS1**	21,525,372,600	13,482,235,967	62.6	30.3	24.1	30.8	14.8
**ALS2**	17,620,068,900	11,688,557,657	66.3	25.4	39.7	22.8	12.2
**ALS3**	23,518,969,200	15,062,468,598	64.0	35.8	24.1	24.8	15.3
**ALS4**	19,429,933,500	12,750,324,647	65.6	37.1	25.8	22.4	14.7
**ALS9**	25,847,743,800	15,956,695,398	61.7	36.7	21.4	27.4	14.4
**ALS10**	18,557,838,300	12,691,363,772	68.4	31.8	25.7	27.8	14.7
**ALS14**	18,327,581,100	12,410,814,921	67.7	43.7	19.7	25.6	11.0
**CTL6**	25,560,552,300	15,739,588,406	61.6	37.3	19.7	26.5	16.5
**CTL8**	24,262,752,600	15,323,900,550	63.2	33.1	22.7	27.9	16.3
**CTL16**	22,605,558,600	14,017,986,644	62.0	28.7	30.1	26.9	14.3
**CTL22**	16,823,823,300	11,171,823,684	66.4	30.2	32.4	25.0	12.4
**CTL23**	18,963,078,300	13,009,938,471	68.6	30.1	43.4	16.3	10.2
**CTL24**	18,073,171,500	12,555,674,242	69.5	30.4	38.4	17.8	13.5
**CTL25**	19,274,686,800	13,257,578,863	68.8	35.5	34.3	17.8	12.4
**CTL27**	18,371,600,100	12,471,242,885	67.9	32.5	33.7	21.3	12.5

Supplementary Figure 1: Sample metrics collected using Picard's CollectRNASeqMetrics command. PF = Passed Filter
