# Supplementary material for: RNAseq Analyses Identify Tumor Necrosis Factor-Mediated Inflammation as a Major Abnormality in ALS Spinal Cord
Source: PLoS One. 2016 Aug 3;11(8):e0160520. doi: 10.1371/journal.pone.0160520 (PMC4972368; doi:10.1371/journal.pone.0160520)
Supplement: S2 Fig — Uniquely aligned reads were used for DeSeq2 and EdgeR analyses. (PDF) [file pone.0160520.s002.pdf]

Supplementary Figure 2

Sample ID	Paired End Reads uniquely aligned to hg19 genes	Paired End Reads Multiply aligned to hg19 genes
**ALS1**	17,079,872	13,923,290
**ALS2**	12,142,430	14,946,592
**ALS3**	22,456,944	20,252,817
**ALS4**	20,074,506	29,925,873
**ALS9**	25,147,130	18,710,778
**ALS10**	16,567,040	22,428,047
**ALS14**	22,785,166	15,660,804
**CTL6**	24,978,566	32,026,378
**CTL8**	21,336,355	31,498,430
**CTL16**	17,091,970	21,962,757
**CTL22**	14,265,193	21,672,506
**CTL23**	16,029,151	16,133,502
**CTL24**	15,524,378	25,106,385
**CTL25**	18,959,037	21,189,291
**CTL27**	16,821,773	20,867,799

Supplementary Figure 2 details the number of paired end reads that aligned either uniquely or multiply to hg19 genes. Uniquely aligned reads were used for DeSeq2 and EdgeR analyses.
