# Supplementary material for: RNAseq Analyses Identify Tumor Necrosis Factor-Mediated Inflammation as a Major Abnormality in ALS Spinal Cord
Source: PLoS One. 2016 Aug 3;11(8):e0160520. doi: 10.1371/journal.pone.0160520 (PMC4972368; doi:10.1371/journal.pone.0160520)
Supplement: S3 Fig — (PDF) [file pone.0160520.s003.pdf]

Supplementary Figure 3

Downregulated DEGs

Top Canonical Pathways	p-Value
Superpathway of Cholesterol Biosynthesis	1.83E-06
Cholesterol Biosynthesis I	5.64E-05
Cholesterol Biosynthesis II	5.64E-05
Cholesterol Biosynthesis III	5.64E-05
Hepatic Fibrosis	5.16E-04
**Top Diseases and Disorders**	**p-Value**
Inflammatory Disease	2.63E-03-3.17E-08
Inflammatory Response	1.91E-03-3.17E-08
Organismal Injury and Abnormalities	2.63E-03-3.17E-08
Renal and Urological Disease	2.63E-03-3.17E-08
Dermatological Diseases and Conditions	1.75E-03-1.07E-07
**"Activated" Upstream Regulators**	**p-Value**
Arsenic trioxide	4.36E-08
Beta-estradiol	1.23E-07
DICER1	1.80E-07
cholesterol	2.94E-07
Lysophosphatidic acid	3.02E-07

Supplementary Figure 3 shows IPA Core Analysis Results for DEGs (18) identified as downregulated in the ALS group by all three analyses.
