# Supplementary material for: RNAseq Analyses Identify Tumor Necrosis Factor-Mediated Inflammation as a Major Abnormality in ALS Spinal Cord
Source: PLoS One. 2016 Aug 3;11(8):e0160520. doi: 10.1371/journal.pone.0160520 (PMC4972368; doi:10.1371/journal.pone.0160520)
Supplement: S4 Fig — Fold change values from Cuffdiff2 are listed. (PDF) [file pone.0160520.s004.pdf]

Supplementary Figure 4

Gene	Fold Change
ABCG1	2.18
APOC1	4.61
APOE	3.25
ASAH1	1.85
BMF	2.73
CAPG	3.00
CD37	2.66
CD84	1.99
CD86	2.01
CEBPA	3.29
CECR1	1.73
CPM	2.32
CPVL	3.40
CTSD	2.35
CTSS	2.36
CXCL16	2.40
DNASE2	1.90
FCGR2B	2.98
GNB4	1.61
GPNMB	5.39
GPRIN3	1.84
HAVCR2	2.08
HLA-DMB	2.76
HLA-DOA	2.32
HLA-DRB1	4.10
HPSE	2.96
IL18	1.98
ITGAX	2.68
KCNJ5	2.43
LILRA2	3.59
LILRB4	2.98
LTA4H	1.65
OTUD1	1.79
PIK3IP1	1.94
PLA2G7	5.59
SELPLG	2.82
SIGLEC8	4.40
SLC37A2	3.28
SLC7A7	3.01
TBXAS1	2.12
TNFAIP2	2.29
WDR91	1.72

Supplementary Figure 4 lists DEGs identified as “upregulated” in the ALS group that were also found in the black module. Fold change values from Cuffdiff2 are listed.
