# Supplementary material for: RNAseq Analyses Identify Tumor Necrosis Factor-Mediated Inflammation as a Major Abnormality in ALS Spinal Cord
Source: PLoS One. 2016 Aug 3;11(8):e0160520. doi: 10.1371/journal.pone.0160520 (PMC4972368; doi:10.1371/journal.pone.0160520)
Supplement: S5 Fig — The empty vector used for the EV group was the same plasmid with TNFAIP2 CDS taken out. (PDF) [file pone.0160520.s005.pdf]

Supplementary Figure 5

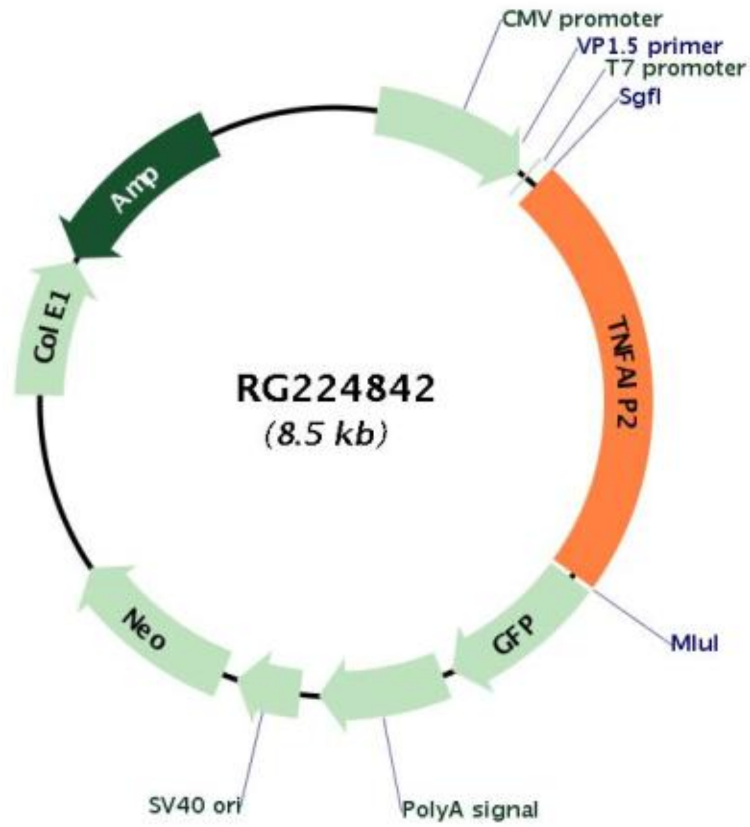

Supplementary Figure 5 shows the TNFAIP2-GFP plasmid used in this experiment. The empty vector used for the EV group was the same plasmid with TNFAIP2 CDS taken out.
