# Supplementary material for: RNAseq Analyses Identify Tumor Necrosis Factor-Mediated Inflammation as a Major Abnormality in ALS Spinal Cord
Source: PLoS One. 2016 Aug 3;11(8):e0160520. doi: 10.1371/journal.pone.0160520 (PMC4972368; doi:10.1371/journal.pone.0160520)
Supplement: S6 Fig — (PDF) [file pone.0160520.s006.pdf]

Supplementary Figure 6

Sienna 4 Module Genes

Top Canonical Pathways	p-Value
Methylglyoxal Degradation VI	2.78E-03
Phosphatidylethanolamine Biosynthesis III	2.78E-03
NGF Signaling	3.29E-03
Branched-chain –keto acid Dehydrogenase Complex	1.11E-02
Phenylethylamine Degradation I	1.11E-02
**Top Diseases and Disorders**	**p-Value**
Developmental Disorder	3.28E-02-7.55E-05
Hereditary Disorder	3.28E-02-7.55E-05
Metabolic Disease	2.44E-02-7.55E-05
Organismal Injury and Abnormalities	4.14E-02-7.55E-05
Renal and Urological Disease	5.55E-03-7.55E-05
**"Activated" Upstream Regulators**	**p-Value**
STAT6	5.19E-04
STAT3/5	5.10E-03
Butylbenzyl phthalate	5.10E-03
Folic acid	6.16E-03
WHSC1L1	7.64E-03

Supplementary Figure 6 shows IPA Core Analysis Results for genes (67) comprising the sienna4 module.
