# Supplementary material for: RNAseq Analyses Identify Tumor Necrosis Factor-Mediated Inflammation as a Major Abnormality in ALS Spinal Cord
Source: PLoS One. 2016 Aug 3;11(8):e0160520. doi: 10.1371/journal.pone.0160520 (PMC4972368; doi:10.1371/journal.pone.0160520)
Supplement: S7 Fig — ALS = amyotrophic lateral sclerosis; CTL = control. (PDF) [file pone.0160520.s007.pdf]

## Supplementary Figure 7

| | Age | Ethnicity | Gender |
|---|---|---|---|
| **Patients** | | | |
| **ALS1** | 70 | Caucasian | Male |
| **ALS2** | 67 | Caucasian | Male |
| **ALS3** | 80 | Caucasian | Female |
| **ALS4** | 57 | Caucasian | Male |
| **ALS9** | 75 | Caucasian | Female |
| **ALS10** | 64 | Caucasian | Female |
| **ALS14** | 61 | Caucasian | Male |
| | | | |
| **Controls** | | | |
| **CTL6** | 80 | African-American | Male |
| **CTL8** | 67 | Caucasian | Female |
| **CTL16** | 66 | Caucasian | Female |
| **CTL22** | 54 | Caucasian | Male |
| **CTL23** | 65 | African-American | Female |
| **CTL24** | 83 | Caucasian | Male |
| **CTL25** | 59 | Caucasian | Male |
| **CTL27** | 84 | Caucasian | Female |

Supplementary Figure 7 shows all sample’s age, ethnicity, and gender information.
