# Supplementary material for: RNAseq Analyses Identify Tumor Necrosis Factor-Mediated Inflammation as a Major Abnormality in ALS Spinal Cord
Source: PLoS One. 2016 Aug 3;11(8):e0160520. doi: 10.1371/journal.pone.0160520 (PMC4972368; doi:10.1371/journal.pone.0160520)
Supplement: S8 Fig — The results approximate scale free topology at .80 correlation. Data are derived from the WGCNA analyses. (PDF) [file pone.0160520.s008.pdf]

Supplementary Figure 8

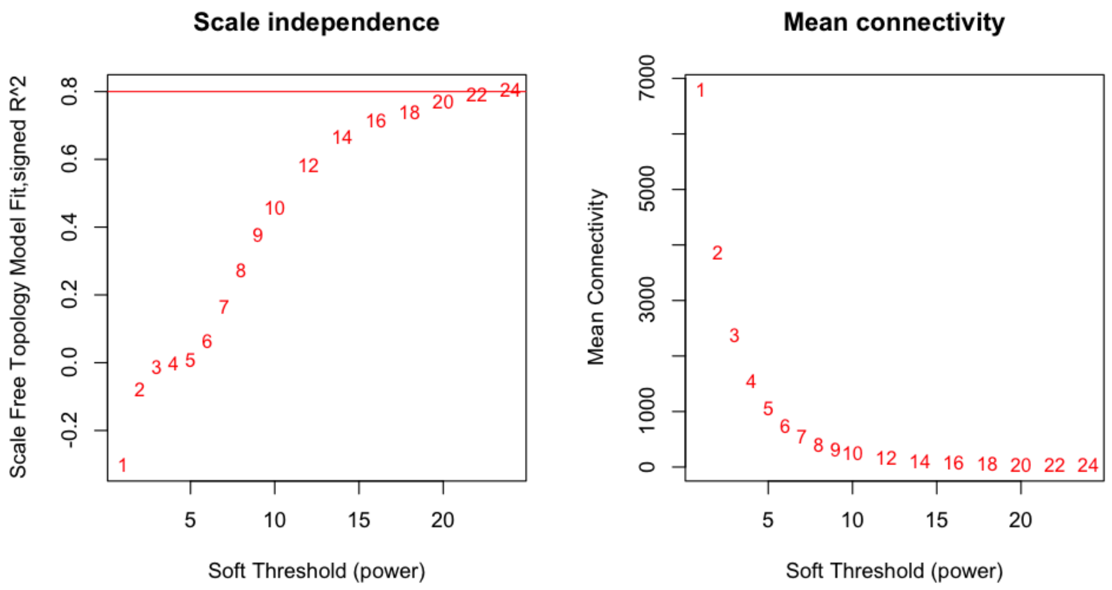

Supplementary Figure 8 shows raising the adjacency matrix to the power of 24 approximates scale free topology at .80.
