# Supplementary material for: RNAseq Analyses Identify Tumor Necrosis Factor-Mediated Inflammation as a Major Abnormality in ALS Spinal Cord
Source: PLoS One. 2016 Aug 3;11(8):e0160520. doi: 10.1371/journal.pone.0160520 (PMC4972368; doi:10.1371/journal.pone.0160520)
Supplement: S1 Note — (PDF) [file pone.0160520.s009.pdf]

Supplementary Note 1

Pre-Processing and Alignment Commands

Trimmomatic Command

```
java -jar trimmomatic-0.32.jar PE -threads 15 -phred33
/Volumes/Drobo_Storage/Raw_Data_and_Trimmomatic_Files/First_Six_Samples_Raw_Data/Ra
w_Seq_Files/ALS1_1_sequence.txt.gz
/Volumes/Drobo_Storage/Raw_Data_and_Trimmomatic_Files/First_Six_Samples_Raw_Data/Ra
w_Seq_Files/ALS1_2_sequence.txt.gz
/Volumes/Drobo_Storage/Raw_Data_and_Trimmomatic_Files/Processed_Samples/Postmortem_
Spinal_Samples/ALS1/ALS1_forward_paired.fq
/Volumes/Drobo_Storage/Raw_Data_and_Trimmomatic_Files/Processed_Samples/Postmortem_
Spinal_Samples/ALS1/ALS1_forward_unpaired.fq
/Volumes/Drobo_Storage/Raw_Data_and_Trimmomatic_Files/Processed_Samples/Postmortem_
Spinal_Samples/ALS1/ALS1_reverse_paired.fq
/Volumes/Drobo_Storage/Raw_Data_and_Trimmomatic_Files/Processed_Samples/Postmortem_
Spinal_Samples/ALS1/ALS1_reverse_unpaired.fq ILLUMINACLIP:TruSeq3-PE-
2.fa:2:30:10:8:true TRAILING:20 MINLEN:50
```

BWA Commands

```
head -5000000 ALS1_forward_paired.fq > 1.txt
head -5000000 ALS1_reverse_paired.fq > 2.txt

bwa aln hg19tr.fa 1.txt > 1.sai
bwa aln hg19tr.fa 2.txt > 2.sai

bwa sampe hg19tr.fa 1.sai 2.sai 1.txt 2.txt > aln.sam

samtools view -Sb aln.sam > aln.bam

samtools sort aln.bam aln_sorted

java -jar picard.jar CollectInsertSizeMetrics I=aln_sorted.bam O=out.metrics
HISTOGRAM_FILE=chartoutput.pdf VALIDATION_STRINGENCY=LENIENT
```

This command will generate a file called “out.metrics” in the picard folder containing mean_insert size and standard deviation values

Subtract the total length of your reads (in our case 2X150, so 300 total) from the mean_insert size – i.e 198-300 = -102 for the Tophat r value.

Tophat Command

```
tophat -p 2 -o /Volumes/Drobo_Storage/Tophat_Output/ALS1_150_ARemoved_AllReads -G
/Users/Bennett_Lab/Desktop/Tuxedo_Suite/Homo_sapiens_UCSC_hg19/Homo_sapiens/UCSC/
hg19/Annotation/Genes/genes.gtf --library-type fr-firststrand -r -102 --mate-std-dev 49 --
transcriptome-index=hg19.transcriptome/hg19tr genome
/Volumes/Drobo_Storage/Raw_Data_and_Trimmomatic_Files/Processed_Samples/Postmortem_
Spinal_Samples/ALS1/ALS1_forward_paired.fq
/Volumes/Drobo_Storage/Raw_Data_and_Trimmomatic_Files/Processed_Samples/Postmortem_
Spinal_Samples/ALS1/ALS1_reverse_paired.fq
```

Collect RNA-Seq Metrics Command

java -jar picard.jar CollectRnaSeqMetrics REF_FLAT=/Volumes/Drobo_Storage/Homo_sapiens_UCSC_hg19/Homo_sapiens/UCSC/hg19/Annotation/Genes/refFlat.txt.gz RIBOSOMAL_INTERVALS= intervallist.txt STRAND_SPECIFICITY=SECOND_READ_TRANSCRIPTION_STRAND INPUT=accepted_hits.bam OUTPUT=Stats

GATK Point Mutation Analysis Commands

STAR --runMode genomeGenerate --genomeDir /Volumes/Drobo_Storage/STARgenome --genomeFastaFiles /Volumes/Drobo_Storage/Homo_sapiens_UCSC_hg19/Homo_sapiens/UCSC/hg19/Sequence/Bowtie2Index/genome.fa --runThreadN 10

STAR --genomeDir /Volumes/Drobo_Storage/STARgenome/ --readFilesIn /Volumes/Drobo_Storage/Raw_Data_and_Trimmomatic_Files/Processed_Samples/ALS1/150Bp_AllReads_Adaptors_Removed/ALS1_forward_paired.fq /Volumes/Drobo_Storage/Raw_Data_and_Trimmomatic_Files/Processed_Samples/ALS1/150Bp_AllReads_Adaptors_Removed/ALS1_reverse_paired.fq --runThreadN 15

STAR --runMode genomeGenerate --genomeDir /Volumes/Drobo_Storage/STARgenome_2ndpass/ --genomeFastaFiles /Volumes/Drobo_Storage/Homo_sapiens_UCSC_hg19/Homo_sapiens/UCSC/hg19/Sequence/Bowtie2Index/genome.fa --sjdbFileChrStartEnd /Volumes/Drobo_Storage/STAR_Alignments/PAT_ALS1/SJ.out.tab --sjdbOverhang 75 --runThreadN 15

STAR --genomeDir /Volumes/Drobo_Storage/STARgenome_2ndpass/ --readFilesIn /Volumes/Drobo_Storage/Raw_Data_and_Trimmomatic_Files/Processed_Samples/ALS1/150Bp_AllReads_Adaptors_Removed/ALS1_forward_paired.fq /Volumes/Drobo_Storage/Raw_Data_and_Trimmomatic_Files/Processed_Samples/ALS1/150Bp_AllReads_Adaptors_Removed/ALS1_reverse_paired.fq --runThreadN 15

java -jar picard.jar AddOrReplaceReadGroups I=Aligned.out.sam O=rg_added_sorted.bam SO=coordinate RGID=ALS1 RGLB=ALS1 RGPL=ILLUMINA RGPU=NextSeq500 RGSM=ALS1

java -Xmx50g -jar picard.jar MarkDuplicates I=rg_added_sorted.bam O=deduped.bam CREATE_INDEX=true VALIDATION_STRINGENCY=SILENT M=output.metrics

java -jar GenomeAnalysisTK.jar -T SplitNCigarReads -R /Volumes/Drobo_Storage/Homo_sapiens_UCSC_hg19/Homo_sapiens/UCSC/hg19/Sequence/Bowtie2Index/genome.fa -I deduped.bam -o split.bam -rf ReassignOneMappingQuality -RMQF 255 -RMQT 60 -U ALLOW_N_CIGAR_READS

java -jar GenomeAnalysisTK.jar -T BaseRecalibrator -R /Volumes/Drobo_Storage/Homo_sapiens_UCSC_hg19/Homo_sapiens/UCSC/hg19/Sequence/Bowtie2Index/genome.fa -I split.bam -knownSites /Volumes/Drobo_Storage/Homo_sapiens_UCSC_hg19/Homo_sapiens/UCSC/hg19/Annotation/Everything_Else/Variation/dbsnp_138.hg19.vcf -nct 14 -o recal_data.table

java -jar GenomeAnalysisTK.jar -T BaseRecalibrator -R /Volumes/Drobo_Storage/Homo_sapiens_UCSC_hg19/Homo_sapiens/UCSC/hg19/Sequence/Bo

wtie2Index/genome.fa -I split.bam -knownSites /Volumes/Drobo_Storage/Homo_sapiens_UCSC_hg19/Homo_sapiens/UCSC/hg19/Annotation/Everything_Else/Variation/dbsnp_138.hg19.vcf -BQSR recal_data.table -nct 14 -o Final_recal_data.table

java -jar GenomeAnalysisTK.jar -T PrintReads -R /Volumes/Drobo_Storage/Homo_sapiens_UCSC_hg19/Homo_sapiens/UCSC/hg19/Sequence/Bowtie2Index/genome.fa -I split.bam -BQSR Final_recal_data.table -o Recal_split.bam

java -jar GenomeAnalysisTK.jar -T HaplotypeCaller -D /Volumes/Drobo_Storage/Homo_sapiens_UCSC_hg19/Homo_sapiens/UCSC/hg19/Annotation/Everything_Else/Variation/dbsnp_138.hg19.vcf -R /Volumes/Drobo_Storage/Homo_sapiens_UCSC_hg19/Homo_sapiens/UCSC/hg19/Sequence/Bowtie2Index/genome.fa -I Recal_split.bam -dontUseSoftClippedBases -stand_call_conf 20.0 -stand_emit_conf 20.0 -o output.vcf

java -jar GenomeAnalysisTK.jar -T VariantFiltration -R /Volumes/Drobo_Storage/Homo_sapiens_UCSC_hg19/Homo_sapiens/UCSC/hg19/Sequence/Bowtie2Index/genome.fa -V output.vcf -window 35 -cluster 3 -filterName FS -filter "FS >30.0" -filterName QD -filter "QD <2.0" -o final_output.vcf

HTSeq Count Command

From within a Tophat Output Folder

samtools sort -n accepted_hits.bam accepted_hits.sorted

samtools view accepted_hits.sorted.bam | htseq-count -s reverse - /Volumes/Drobo_Storage/Homo_sapiens_UCSC_hg19/Homo_sapiens/UCSC/hg19/Annotation/Genes/genes.gtf > PAT_ALS1_Count.txt

Cufflinks, Cuffmerge, and Cuffdiff Commands

Cufflinks Command

cufflinks -o /Volumes/Drobo_Storage/Cufflinks_Cuffdiff_Output/Postmortem_Spinals_Cufflinks_Output_No_M/PAT_ALS1_no_novel_highdata1 --library-type fr-firststrand -p 1 -G /Users/Bennett_Lab/Desktop/Tuxedo_Suite/Homo_sapiens_UCSC_hg19/Homo_sapiens/UCSC/hg19/Annotation/Genes/genes.gtf -b /Users/Bennett_Lab/Desktop/Tuxedo_Suite/Homo_sapiens_UCSC_hg19/Homo_sapiens/UCSC/hg19/Sequence/Bowtie2Index/genome.fa -M /Users/Bennett_Lab/Desktop/Tuxedo_Suite/Homo_sapiens_UCSC_hg19/Homo_sapiens/UCSC/hg19/Sequence/Bowtie2Index/rRNA.tRNA.Mt.gtf -u --compatible-hits-norm --max-bundle-frags 999999999 /Volumes/Drobo_Storage/Tophat_Output/Postmortem_Spinal_Samples/PAT_ALS1_150_ARemoved_AllReads/accepted_hits.bam

Cuffmerge Command

cuffmerge -p 14 -o CTLvPAT_M -g /Users/Bennett_Lab/Desktop/Tuxedo_Suite/Homo_sapiens_UCSC_hg19/Homo_sapiens/UCSC/hg19/Annotation/Genes/genes.gtf -s

/Users/Bennett_Lab/Desktop/Tuxedo_Suite/Homo_sapiens_UCSC_hg19/Homo_sapiens/UCSC/hg19/Sequence/WholeGenomeFasta/genome.fa assemblies.txt

Cuffdiff2 Command

```
cuffdiff --library-type fr-firststrand -o /Volumes/Drobo_Storage/PATvCTL_No_M_compared -p 14 -b
/Users/Bennett_Lab/Desktop/Tuxedo_Suite/Homo_sapiens_UCSC_hg19/Homo_sapiens/UCSC/hg19/Sequence/Bowtie2Index/genome.fa -u -L CTL,PAT -M
/Users/Bennett_Lab/Desktop/Tuxedo_Suite/Homo_sapiens_UCSC_hg19/Homo_sapiens/UCSC/hg19/Sequence/Bowtie2Index/rRNA.tRNA.Mt.gtf -max-bundle-frags 999999999 -v
/Volumes/Drobo_Storage/CTLvPAT_No_M/merged.gtf
/Volumes/Drobo_Storage/Tophat_Output/CTL_ALS6_150_ARemoved_AllReads/accepted_hits.bam,/Volumes/Drobo_Storage/Tophat_Output/CTL_ALS8_150_ARemoved_AllReads/accepted_hits.bam,/Volumes/Drobo_Storage/Tophat_Output/CTL_ALS16_150_ARemoved_AllReads/accepted_hits.bam,/Volumes/Drobo_Storage/Tophat_Output/CTL_ALS22_150_ARemoved_AllReads/accepted_hits.bam,/Volumes/Drobo_Storage/Tophat_Output/CTL_ALS23_150_ARemoved_AllReads/accepted_hits.bam,/Volumes/Drobo_Storage/Tophat_Output/CTL_ALS24_150_ARemoved_AllReads/accepted_hits.bam,/Volumes/Drobo_Storage/Tophat_Output/CTL_ALS25_150_ARemoved_AllReads/accepted_hits.bam,/Volumes/Drobo_Storage/Tophat_Output/CTL_ALS27_150_ARemoved_AllReads/accepted_hits.bam
/Volumes/Drobo_Storage/Tophat_Output/PAT_ALS1_150_ARemoved_AllReads/accepted_hits.bam,/Volumes/Drobo_Storage/Tophat_Output/PAT_ALS2_150_ARemoved_AllReads/accepted_hits.bam,/Volumes/Drobo_Storage/Tophat_Output/PAT_ALS3_150_ARemoved_AllReads/accepted_hits.bam,/Volumes/Drobo_Storage/Tophat_Output/PAT_ALS4_150_ARemoved_AllReads/accepted_hits.bam,/Volumes/Drobo_Storage/Tophat_Output/PAT_ALS9_150_ARemoved_AllReads/accepted_hits.bam,/Volumes/Drobo_Storage/Tophat_Output/PAT_ALS10_150_ARemoved_AllReads/accepted_hits.bam,/Volumes/Drobo_Storage/Tophat_Output/PAT_ALS14_150_ARemoved_AllReads/accepted_hits.bam
```

Calculating p-values using Benjamini-Hochberg correction

#Copy the p-value column in gene_exp.diff output file. Paste them into first column of Excel file, and save as “All_P.csv”. Open R, then…#

```
Data <- read.csv(file="All_P.csv",header=TRUE,stringsAsFactors=FALSE)
FDR <- p.adjust(Data[,1],method="BH")
write.csv(FDR, file="FDR.csv")
```

#Paste these values back into the gene_exp.diff file. These are FDR corrected p values.

DeSeq2 Commands

```
source("http://bioconductor.org/biocLite.R")
biocLite("DESeq2")
biocLite("pasilla")
library(DESeq2)
library(pasilla)
library(Biobase)
setwd("/Users/Bennett_Lab/Desktop/DESeq2/Individual_Count_Files_Postmortem")
getwd()

directory = "/Users/Bennett_Lab/Desktop/DESeq2/Individual_Count_Files_Postmortem/"
sampleFiles <- grep("PAT",list.files(directory),value=TRUE)
```

```
sampleCondition <- sub("(.*PAT).*","\\1",sampleFiles)
sampleTable <- data.frame(sampleName = sampleFiles, fileName = sampleFiles, condition =
sampleCondition)
ddsHTSeq <- DESeqDataSetFromHTSeqCount(sampleTable = sampleTable, directory =
directory, design = ~ condition)
dds <- DESeq(ddsHTSeq)
res <- results(dds)
write.csv(res, file="Res.csv")
```

edgeR Commands

```
setwd("/Users/Bennett_Lab/Desktop/EdgeR/Postmortem Spinal Samples/")
source("http://bioconductor.org/biocLite.R")
biocLite("edgeR")
library(edgeR)

x <- read.delim("EdgeR.txt",row.names="Symbol")
group <- factor(c(2,2,2,2,2,2,2,1,1,1,1,1,1,1,1))
y <- DGEList(counts=x,group=group)
keep <- rowSums(cpm(y)>1) >= 7
y <- y[keep,]
y$samples$lib.size <- colSums(y$counts)
y$samples
y <- calcNormFactors(y)
y <- estimateCommonDisp(y)
y <- estimateTagwiseDisp(y)
et <- exactTest(y)
topTags(et)
et$table
write.csv(et$table, "DEGList.csv")

#Copy the Pvalue column in the DEGList.csv output file. Paste them into first column of Excel file,
and save as "All_P.csv". Open R, then…#

Data <- read.csv(file="All_P.csv",header=TRUE,stringsAsFactors=FALSE)
FDR <- p.adjust(Data[,1],method="BH")
write.csv(FDR, file="FDR.csv")


#Paste these adjusted p values back into the DEGList.csv file. These are FDR corrected p
values.
```

WGCNA Commands

```
setwd("/Users/Bennett_Lab/Desktop/WGCNA")
getwd()
source("http://bioconductor.org/biocLite.R")
biocLite("impute")
install.packages("WGCNA")
library(WGCNA);

options(stringsAsFactors = FALSE)

ALLOW_WGCNA_THREADS=6
```

```
lnames=load(file="AllSubjects-data.input.Rdata") # This loads the variables datExpr (including
information from “All_Sample_Data.csv”) and datTraits (including information from datTraits.csv).
####

powers = c(c(1:10), seq(from = 12, to=24, by=2))

datExpr[, c(1:13301)] <- sapply(datExpr[, c(1:13301)], as.numeric)

sft= pickSoftThreshold(datExpr, powerVector = powers, verbose = 5, networkType ="signed",
corFnc= "bicor", corOptions=list(maxPOutliers=0.1))

sizeGrWindow(9, 5)
par(mfrow = c(1,2));
cex1 = 0.9;

plot(sft$fitIndices[,1], -sign(sft$fitIndices[,3])*sft$fitIndices[,2], xlab="Soft Threshold
(power)",ylab="Scale Free Topology Model Fit,signed R^2",type="n", main = paste("Scale
independence")); text(sft$fitIndices[,1], -sign(sft$fitIndices[,3])*sft$fitIndices[,2],
labels=powers,cex=cex1,col="red");

abline(h=0.80,col="red")

plot(sft$fitIndices[,1], sft$fitIndices[,5], xlab="Soft Threshold (power)",ylab="Mean Connectivity",
type="n", main = paste("Mean connectivity")) text(sft$fitIndices[,1], sft$fitIndices[,5],
labels=powers, cex=cex1,col="red")

adjacency = adjacency(datExpr, power=24, corFnc="bicor", type="signed", corOptions = "use =
'p', maxPOutliers = 0.1")

TOM= TOMsimilarity(adjacency)
dissTOM = 1-TOM

geneTree = hclust(as.dist(dissTOM), method = "average");
sizeGrWindow(12,9)
plot(geneTree, xlab="", sub="", main = "Gene clustering on TOM-based dissimilarity", labels =
FALSE, hang = 0.04);

minModuleSize = 30;

dynamicMods = cutreeDynamic(dendro = geneTree, distM = dissTOM, deepSplit = 2,
pamRespectsDendro = FALSE, minClusterSize = minModuleSize);

table(dynamicMods)
dynamicColors = labels2colors(dynamicMods)
table(dynamicColors)

sizeGrWindow(8,6)
plotDendroAndColors(geneTree, dynamicColors, "Dynamic Tree Cut", dendroLabels = FALSE,
hang = 0.03, addGuide = TRUE, guideHang = 0.05, main = "Gene dendrogram and module
colors")

MEList = moduleEigengenes(datExpr, colors = dynamicColors)
MEs = MEList$eigengenes

MEDiss = 1-cor(MEs);
METree = hclust(as.dist(MEDiss), method = "average");
```

```
sizeGrWindow(7, 6)

plot(METree, main = "Clustering of module eigengenes", xlab = "", sub = "")
MEDissThres = 0.25
abline(h=MEDissThres, col = "red")

merge = mergeCloseModules(datExpr, dynamicColors, cutHeight = MEDissThres, verbose = 3)

mergedColors = merge$colors;

mergedMEs = merge$newMEs;

plotDendroAndColors(geneTree, cbind(dynamicColors, mergedColors), c("Dynamic Tree Cut",
"Merged dynamic"), dendroLabels = FALSE, hang = 0.03, addGuide = TRUE, guideHang = 0.05)

moduleColors = mergedColors

colorOrder = c("grey", standardColors(50));

moduleLabels = match(moduleColors, colorOrder)-1;

MEs = mergedMEs;

nGenes = ncol(datExpr);

nSamples = nrow(datExpr);

MEs0 = moduleEigengenes(datExpr, moduleColors)$eigengenes

MEs = orderMEs(MEs0)

moduleTraitCor = cor(MEs, datTraits, use = "p");

moduleTraitPvalue = corPvalueStudent(moduleTraitCor, nSamples);

sizeGrWindow(10,6)

textMatrix = paste(signif(moduleTraitCor, 2), "\n(", signif(moduleTraitPvalue, 1), ")", sep = "");
dim(textMatrix) = dim(moduleTraitCor) par(mar = c(6, 8.5, 3, 3));

labeledHeatmap(Matrix = moduleTraitCor, xLabels = names(datTraits), yLabels = names(MEs),
ySymbols = names(MEs), colorLabels = FALSE, colors = greenWhiteRed(50), textMatrix =
textMatrix, setStdMargins = FALSE, cex.text = 0.5, zlim = c(-1,1), main = paste("Module-trait
relationships"))

##### This is for modular membership scores #####
datKME = signedKME(datExpr, MEs, outputColumnName = "kME")
write.csv(datKME, file="datKME.csv")

##### This is for gene significance scores #####
Disease = as.data.frame(datTraits$Disease.Status)
GS.Disease = as.numeric(cor(datExpr, Disease, use = "p"))
write.csv(GS.Disease, file="GS.Disease.csv")

##### This is for intramodular connectivity scores #####
```

```
IMConnectivity = intramodularConnectivity(adjacency, moduleColors, scaleByMax = FALSE)

write.csv(IMConnectivity, file="IMConnectivity.csv")
```

To retrieve module genes for IPA Core Analyses:

```
module = "black"
probes = names(datExpr)
inModule = (moduleColors==module);
modProbes = probes[inModule];
```
